# Tyrosine-modifying glycosylation by *Yersinia* effectors

**DOI:** 10.1016/j.jbc.2024.107331

**Published:** 2024-05-02

**Authors:** Silvia Schneider, Christophe Wirth, Thomas Jank, Carola Hunte, Klaus Aktories

**Affiliations:** 1 Faculty of Medicine, Institute for Experimental and Clinical Pharmacology and Toxicology, University of Freiburg, Freiburg, Germany; 2 Faculty of Medicine, Institute for Biochemistry and Molecular Biology, ZBMZ, University of Freiburg, Freiburg, Germany; 3 Centre for Biological Signalling Studies (BIOSS), University of Freiburg, Freiburg, Germany; 4 CIBSS – Centre for Integrative Biological Signalling Studies, University of Freiburg, Freiburg, Germany

**Keywords:** glycosyltransferase, tyrosine glycosylation, toxin, Rho GTPases, *Photorhabdus asymbiotica* toxin, *Yersinia ruckeri*, *Yersinia enterocolitica*, *Yersinia kristensenii*, *Clostridioides difficile*

## Abstract

Mono-O-glycosylation of target proteins by bacterial toxins or effector proteins is a well-known mechanism by which bacteria interfere with essential functions of host cells. The respective glycosyltransferases are important virulence factors such as the *Clostridioides difficile* toxins A and B. Here, we describe two glycosyltransferases of *Yersinia* species that have a high sequence identity: YeGT from the zoonotic pathogen *Yersinia enterocolitica* and YkGT from the murine pathogen *Yersinia kristensenii*. We show that both modify Rho family proteins by attachment of GlcNAc at tyrosine residues (Tyr-34 in RhoA). Notably, the enzymes differed in their target protein specificity. While YeGT modified RhoA, B, and C, YkGT possessed a broader substrate spectrum and glycosylated not only Rho but also Rac and Cdc42 subfamily proteins. Mutagenesis studies indicated that residue 177 is important for this broader target spectrum. We determined the crystal structure of YeGT shortened by 16 residues N terminally (sYeGT) in the ligand-free state and bound to UDP, the product of substrate hydrolysis. The structure assigns sYeGT to the GT-A family. It shares high structural similarity to glycosyltransferase domains from toxins. We also demonstrated that the 16 most N-terminal residues of YeGT and YkGT are important for the mediated translocation into the host cell using the pore-forming protective antigen of anthrax toxin. Mediated introduction into HeLa cells or ectopic expression of YeGT and YkGT caused morphological changes and redistribution of the actin cytoskeleton. The data suggest that YeGT and YkGT are likely bacterial effectors belonging to the family of tyrosine glycosylating bacterial glycosyltransferases.

The glycosylation of host proteins is a well-known mechanism by which bacterial pathogens manipulate eukaryotic target cells with the help of toxins to circumvent defense mechanisms of the host and/or to optimize their replication niche (1, 2, 3). Well-studied examples of this type of toxins are large clostridial glycosylating toxins, including *Clostridioides difficile* (former name *Clostridium difficile*) toxin A (TcdA) and B (TcdB) (4, 5), and *Paeniclostridium sordellii* (also known as *Clostridium sordellii*) hemorrhagic and lethal toxins (6, 7). Using UDP-glucose as a sugar donor, these toxins glucosylate Rho/Ras GTPases at a crucial threonine residue (*e.g.*, RhoA at Thr-37 and Rac or Ras at Thr-35) (4, 8, 9), thereby inhibiting effector interactions of these proteins (10, 11, 12). Related toxins like *Clostridium novyi* α-toxin (13) or *Clostridium perfringens* TpeL (14, 15) modify Rho/Ras proteins by attachment of GlcNAc at same target amino acid residues.

The previously identified toxin PaTox, which is produced by the entomopathogenic and emergent human pathogenic bacterium *Photorhabdus asymbiotica*, modifies Rho proteins by GlcNAcylation at Tyr-34 of RhoA, similarly resulting in inhibition of switch functions of Rho proteins (16). The glycosylation of tyrosine residues by bacterial toxin is still little explored. Glucosylation of Thr-37 by *C. difficile* toxins A and B or GlcNAcylation of Tyr-34 of RhoA by PaTox inhibits the activation of Rho proteins by guanine nucleotide exchange factors (GEFs), prevents the action of GTPase-activating proteins and blocks the productive interaction with Rho effectors (16, 17).

PaTox is a multifunctional toxin. In addition to its glucosyltransferase activity, it harbors a deamidase activity involved in persistent activation of heterotrimeric G_i_ and G_q_ proteins (16). Thus, the overall structure of PaTox largely differs from that of large clostridial glucosylating toxins; however, their glycosyltransferase domains are similar (4, 16). Several crystal structures of glycosyltransferase domains from PaTox (PaTox^G^) (16) and large clostridial toxins have been determined (18, 19, 20, 21, 22), exhibiting high similarity of their catalytic cores. Both toxin types share the structure of GT-A family glycosyltransferases and possess the typical di-aspartate (DXD) motif (23, 24), involved in coordination of divalent cation (manganese in most structures), a WIG motif, involved in interaction with the uracil moiety of the activated sugar donor, and several highly conserved catalytic amino acids (1, 16, 18, 25). Besides the catalytic core, peripheral subdomains largely differ between large clostridial toxins (*e.g.*, TcdA and TcdB) and PaTox, which is likely to be important for substrate interaction. Notably, both PATOX and clostridial toxins are retaining glycosyltransferases and belong according to Carbohydrate-Active Enzyme (CAZy) database to GT-families 32 and 44, respectively (http://www.cazy.org/GlycosylTransferases.html). Recently, it was shown that *Yersinia ruckeri*, a fish pathogen responsible for the enteric red mouth disease of salmonid fish species (*e.g.*, trout), produces a retaining glycosyltransferase antifeeding prophage 18 (Afp18), which modifies RhoA in Tyr-34 by GlcNAcylation similarly as reported for PaTox (26). In zebrafish embryos, Afp18 causes depolymerization of actin stress fibers, blocks cytokinesis, actin-dependent motility, and cell blebbing, eventually abrogating gastrulation (26). *Y. ruckeri* Afp18 appears to be a bacterial effector protein and is probably translocated into target cells by a phage-derived needle-like complex called Afp, which shares key characteristics with type VI secretion systems (T6SS), R-type pyocins and the *Photorhabdus* virulence cassette (27, 28, 29).

The glycosylating activity of Afp18 found in *Y*. *ruckeri* prompted us to study whether glycosylating toxins/effectors are produced by other species of *Yersinia*. Here, we report that *Yersinia enterocolitica* and *Yersinia kristensenii* encode glycosyltransferase toxins/effectors. We characterized the enzymes and determined the crystal structure of *Y. enterocolitica* GlcNAc transferase.

## Results

### Glycosyltransferases in *Yersinia* species

To identify glycosyltransferase toxins or effectors in *Yersinia* species, we compared the sequences of the glycosyltransferase domains of Afp18, *P. asymbiotica* PaTox^G^ and *C. difficile* TcdB with sequences of putative proteins from various *Yersinia* species in the NCBI Protein database.

Initially, this search resulted in two sequences of hypothetical glycosyltransferases. The first, found in the pathogen *Y*. *enterocolitica* (strain Y2wildboar1B) and comprising 279 residues, was initially described in the database with accession number WP_076706569.1 (hereinafter referred to as sYeGT). A second sequence of 313 residues (Acc.No. WP_049562392) was found in the mouse pathogen *Y*. *kristensenii* strain FCF221 (hereinafter referred to as YkGT). Later, it was noticed that a longer version of sYeGT, with 16 additional residues at the N terminus, exists in the databank sharing the same amino acid length with YkGT (here termed YeGT). This protein is meanwhile listed in the database under Acc.No. WP_139336879.

The amino acid sequences of the long version of YeGT and of YkGT are ∼96% identical between each other (Fig. S1). YeGT and YkGT are ∼78% identical with Afp18 from *Y. ruckeri*, ∼69% with PaTox and only ∼20% with TcdB. Noteworthy, typical amino acid motifs of bacterial glycosyltransferases like the DxD and the WIG motif were conserved. Compared to YeGT and YkGT, the shorter form of YeGT (sYeGT) allowed obtaining high yield of recombinantly expressed and purified protein and thus, we used this construct for most analyses.

### Crystal structure of YeGT

In order to characterize the mechanism of operation of these proteins and to shed light onto their architecture, we initiated crystallization trials with different constructs. sYeGT was crystallized in the absence of ligands and in the presence of UDP-GlcNAc and manganese chloride. Interestingly, in the absence of ligands, sYeGT crystals belonging to space group P4_3_2_1_2 appeared rapidly and diffracted X-rays up to 2.3-Å resolution while in the presence of UDP-GlcNAc, P2_1_ crystals were visible only after 8 weeks but diffracted better, with a resolution up to 1.12 Å (Table 1). Both crystal forms diffracted X-rays anisotropically. After accurate determination of twinning parameters, the phase problem of the ligand bound structure was solved by molecular replacement and refined to final R and R_free_ values of 15.3 and 16.9, respectively. This structure was consequently used to support molecular replacement and model building of the ligand free structure, which was refined to final R and R_free_ values of 20.4 and 24.5, respectively. The atomic coordinates and structure factors of the ligand bound (code 8OVS) and of the ligand free (8OVT) YeGT were deposited in the Protein Data Bank (http://wwpdb.org/).

**Table 1:** Data collection and refinement statistics

Dataset	YeGT	YeGT
	UDP bound state (PDB code 8OVS)	Ligand free (PDB code 8OVT)
Data collection		
Space group	P *2*_1_	P *4*_3_*2*_1_*2*
Cell dimensions		
*a*, *b*, *c* (Å)	47.13, 114.55, 69.37	156.85, 156.85, 156.96
*α, β, γ* (°)	90.00, 89.99, 90.00	90.00, 90.00, 90.00
Number of crystals used for data collection	1	1
Wavelength	1.0000	1.0000
Resolution range (Å)	47.1–1.12 (1.22–1.12)	49.06–2.30 (2.52–2.30)
Resolution limits along a∗, b∗, c∗ (Å)	1.30, 1.12, 1.19	2.84, 2.84, 2.21
Number of unique reflections	213,705 (10,686)	57,009 (2860)
*R*_merge_	0.132 (2.038)	0.188 (2.598)
*R*_*pim*_	0.023 (0.479)	0.045 (0.580)
*I*/σ*I*	18 (1.7)	17.1 (1.8)
*CC*_*1/2*_	0.999 (0.390)	1.000 (0.850)
Completeness (%)		
Spherical	76.6 (17.5)	66.2 (14.0)
Ellipsoidal	92.7 (47.5)	96.4 (83.2)
Multiplicity	30.3 (18.4)	38.6 (40.1)
Refinement		
Resolution (Å)	47.1–1.12	49.1–2.3
No. reflections used in refinement	202,936	56,974
*R*_work_/*R*_free_	15.3/16.9	20.4/24.5
No. atoms		
Protein	4558	9188
Ligands and ions	136	32
Water	872	220
*B* factors		
Protein	17.54	62.16
Ligand	21.03	71.50
Water	33.30	52.66
r.m.s.d.		
Bond lengths (Å)	0.006	0.002
Bond angles (°)	1.196	0.419
Ramachandran plot (%)		
Favored	99.07	97.19
Allowed	0.93	2.82
Outliers	0	0

The asymmetric unit of the ligand bound sYeGT crystal comprises two molecules. Each protomer includes the complete N-terminal part of the construct but lacks 14 residues at the C-terminal end. In addition, a 10-residues long stretch (residues 258–267) was not resolved in the electron density. The ligand-free structure features four molecules in the asymmetric unit. All protomers show clear features in the electron density map for residues 258 to 267, so that they could be included in the structure. Similar to the ligand-bound structure, the C-terminal residues were not resolved and not included in the final structure.

**Figure 1:**
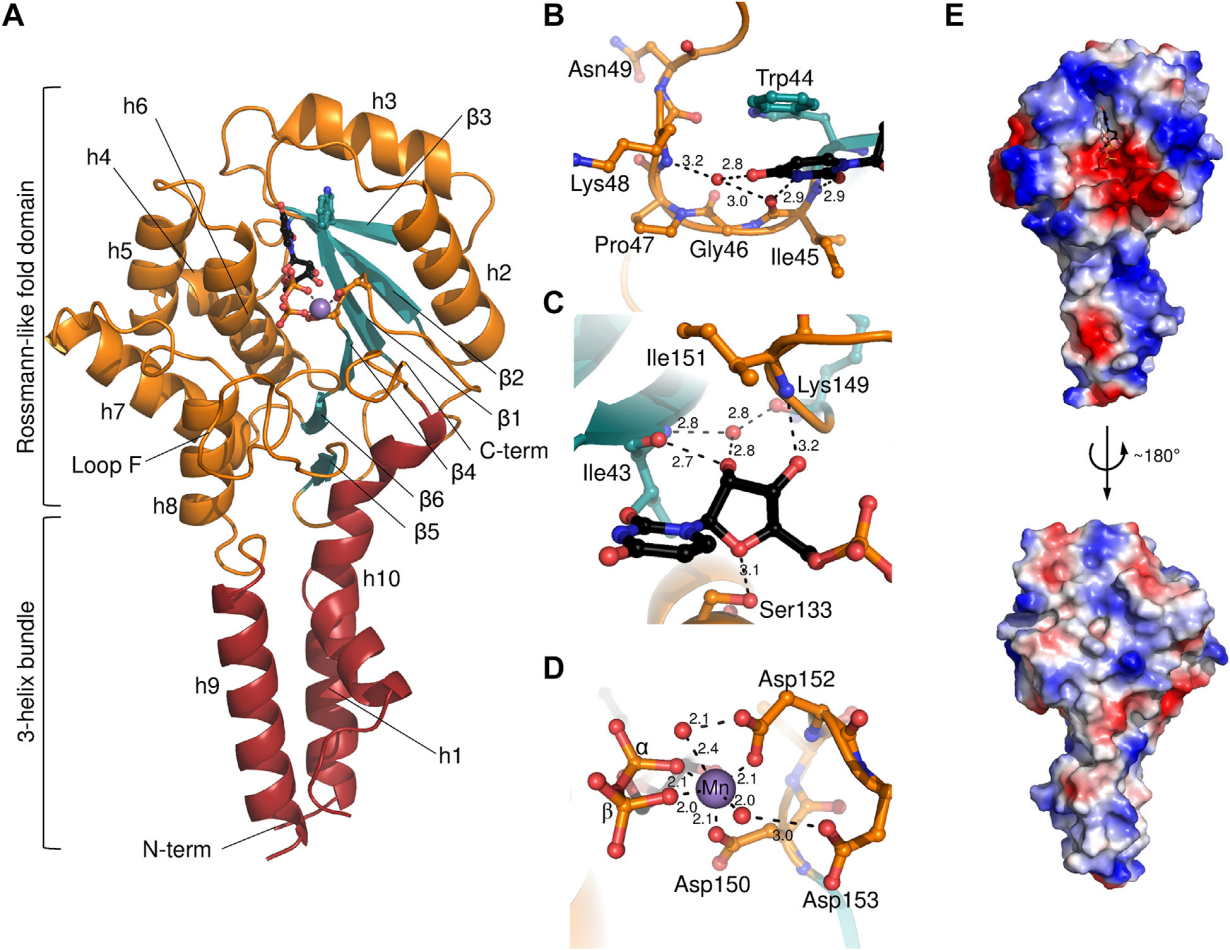
**Crystal structure of sYeGT bound to UDP.***A*, cartoon representation of the overall structure of sYeGT. For the Rossmann-like fold domain, the loops and helices are colored in *orange* and the β-sheets in *teal* while the three-helix bundle is colored *red*. Secondary structure elements are numbered (h for helix, β for β-strand) and the N and C termini as well as Loop F (see text) are indicated. UDP is represented as *black balls* and *sticks*, the manganese ion is represented as a *violet sphere* and the coordinating residues of the DxD motif, as well as the tryptophan forming a stacking interaction with the uridine moiety, are show in *sticks* representation. *B*−*D*, residues (*orange* or *teal sticks* representation) involved in the UDP (*black*) coordination at the uridine (*B*), the ribose (*C*), and the diphosphate (*D*) moieties. Distances are indicated in Ångströms. *E*, surface electrostatic calculation showing the strongly charged surface on the side where the nucleotide binds (*top*) in contrast to the back side of the molecule (*bottom*), where the surface is notably more neutral.

sYeGT has a cone-shaped structure comprised of a globular domain with a Rossmann-like fold on top of a three-helix bundle (Fig. 1*A*). The catalytically active domain is the Rossmann-like fold domain, which features a 6-stranded antiparallel β-sheet flanked by 7 α-helices. The single Rossmann-like fold domain and its characteristics clearly assign sYeGT to the GT-A family of glycosyltransferases.

The structure of sYeGT shows highest similarity to PaTox^G^ with a z-score of 37.8 and an r.m.s.d. of 1.1 Å for 264 aligned Cα atoms (Table 2). PaTox^G^ and sYeGT share the same cone-shaped structures with a Rossmann-like fold domain on top of a three-helix bundle. Further structural homology exists with the glycosyltransferases of large toxins from *Clostridioides* and *Clostridium* species (4, 18, 19, 20) and their homolog from *Yersinia mollaretii* (30). A third group of proteins structurally aligning well with sYeGT comprises *Salmonella* effector proteins (31). Notably, for both large clostridial-type toxins and *Salmonella* effector proteins, the structural similarity is limited to the Rossmann-like fold domain. Overall, sYeGT shares structural similarity with other glycosyltransferase toxins, but shows, together with its close homolog PaTox^G^, a specific 3-helix bundle.

**Table 2:** Structures similar to sYeGT with a z-score higher than 15 as identified by the DALI server (54)

	PDB	Z-score^a^	RMSD	Lali	Nres	% Id	Protein	Organism
1	4mix	37.8	1.1	264	277	70	Glycosyltransferase from PaTox	*Photorhabdus asymbiotica*
2	2bvl	22.0	4.6	218	543	22	Catalytic domain of Toxin B (TcdB)	*Clostridioides difficile*
	6oq7	22.1	4.7	219	541	22		
3	2vkd	21.3	4.7	218	542	22	Catalytic domain of lethal toxin	*Clostridium sordelli*
4	6rth	19.8	2.7	201	504	22	Glycosyltransferase from YGT	*Yersinia mollaretii*
5	4r04	19.3	4.1	222	1793	21	Catalytic domain of Toxin A (TcdA)	*C. difficile*
6	2vk9	17.6	4.0	221	540	23	Catalytic domain of the α-toxin	*C. difficile*
7	5h5y	17.3	3.3	206	286	16	Non-LEE encoded effector protein (NleB)	*Escherichia coli*
8	5h60	17.0	3.3	210	304	16	*Salmonella* effector SseK1	*Salmonella typhimurium*
9	6aci	16.7	3.4	210	304	17	T3SS secreted effector NleB homolog	*E. coli*
10	5h63	16.6	3.1	205	309	19	*Salmonella* effector SseK2	*S. typhimurium*
11	6dus	16.5	3.4	210	310	17	*Salmonella* effector SseK3	*S. typhimurium*
12	3jsz	16.4	3.5	229	520	16	Glycosyltransferase Lgt1	*Legionella pneumophila*
	2wzf	16.4	3.5	228	515	15		

a Z-score represents the statistical significance of the structural alignment between sYeGT and the related proteins; RMSD: Root mean square deviation of equivalent Cα-atoms; lali: number of structurally aligned residues; nres: total number of residues in the hit structure; %id: percentage of identical over the total number of aligned residues when aligning the sYeGT and hit structures.

### The nucleotide binding site

In the high-resolution structure of sYeGT, the difference electron density map enabled unambiguous identification of the ligand UDP (Fig. S2). Although sYeGT was crystallized in the presence of the substrate UDP-GlcNAc and Mn^+2^, only the UDP moiety was visible in the electron density map suggesting that the sugar nucleotide had been hydrolyzed during the crystallization process. The uracil moiety is mainly stabilized by residues from the WIG-motif (residues 44–46). In detail, Trp-44 forms a stacking interaction with the purine base, and backbone atoms of Ile-45 and Lys-48 form hydrogen bonds with the polar side of the base (Fig. 1*B*). Noteworthy, Gly-46 from the WIG-motif and Pro-47 form a kink allowing residues Trp-44 to Lys-48 to encompass the uridine moiety of the nucleotide. The ribose moiety is stabilized by hydrogen bonds to main chain atoms from residues Ile-43 and Ile-151 and to the gamma-oxygen from Ser-133 (Fig. 1*C*). Furthermore, the main chain carbonyl atom of Lys-149 and the nitrogen atom from Ile-43 together bind a water molecule, which also stabilizes the ribose moiety by mediating a hydrogen bond to the hydroxyl group of carbon 2´. The manganese ion is coordinated on one side by the pyrophosphate moiety of UDP and on the other side by the carboxylic groups of Asp-150 and Asp-152, the residues of the DxD motif, (Fig. 1*D*). Notably, a third aspartate residue, Asp-153, is indirectly involved in the manganese coordination *via* a water molecule. In the structure, a Cd^2+^ ion with partial occupancy is bound to the diphosphate group. Cd^2+^ was present in the crystallization buffer. The ion does not interfere with the nucleotide binding by the protein as it is positioned at the opposite side of the diphosphate moiety but its partial presence, as well as that of their hydration shell, limits the analysis of additional water molecules coordinating the diphosphate moiety.

Surface electrostatics analysis revealed that the side of sYeGT, at which the nucleotide binds, is particularly charged while the opposite side of the protein is more electroneutral (Fig. 1*E*). The nucleotide-binding cleft comprises a strongly negatively charged area, ideal to bind the divalent cation, which coordinates the diphosphate, and a strongly positively charged area to which the uridine moiety binds. Overall, the electronegative patch is surrounded by a positively charged rim, which extends from the Rossmann-like fold to the 3-helix bundle.

Superimposition of the ligand-free and ligand bound sYeGT structures showed no major conformational change upon nucleotide binding (Fig. S3). Both structures superimpose with r.m.s.d. values ranging from 0.47 to 0.53 for all C-alpha atoms depending which protomers of the two structures were aligned. Yet, local differences between the two structures were noted. Residues 257 to 267, which are resolved in the ligand-free structure only, form a loop that connects two helices of the 3-helix bundle. At the C terminus, the last resolved residues (294–301) are covering the empty nucleotide-binding site in the ligand free structure, while they are pointing outward in the UDP-bound sYeGT structure. In the ligand free structure, Asp-298 stabilizes the covering position of the C terminus over the unoccupied nucleotide binding side, as it forms a salt bridge with Lys-53, a residue located close to the uridine base binding site. The conformation observed in the ligand-bound structure could be enforced by interactions at the crystal lattice interface. In particular Cys-294, Asp-298, and His-299 are, together with residues Glu-207 and His-211 from the other protomer in the asymmetric unit, involved in the coordination of two Cd^2+^ ions. Another notable feature is the loop flanking the nucleotide binding site (loop F, residues 171–188), which is bent toward the nucleotide when the latter is present. Residues of loop F might play a role in sugar coordination, substrate protein specificity or glycosyltransferase activity.

### Effects of YeGT in eukaryotic cells

**Figure 2:**
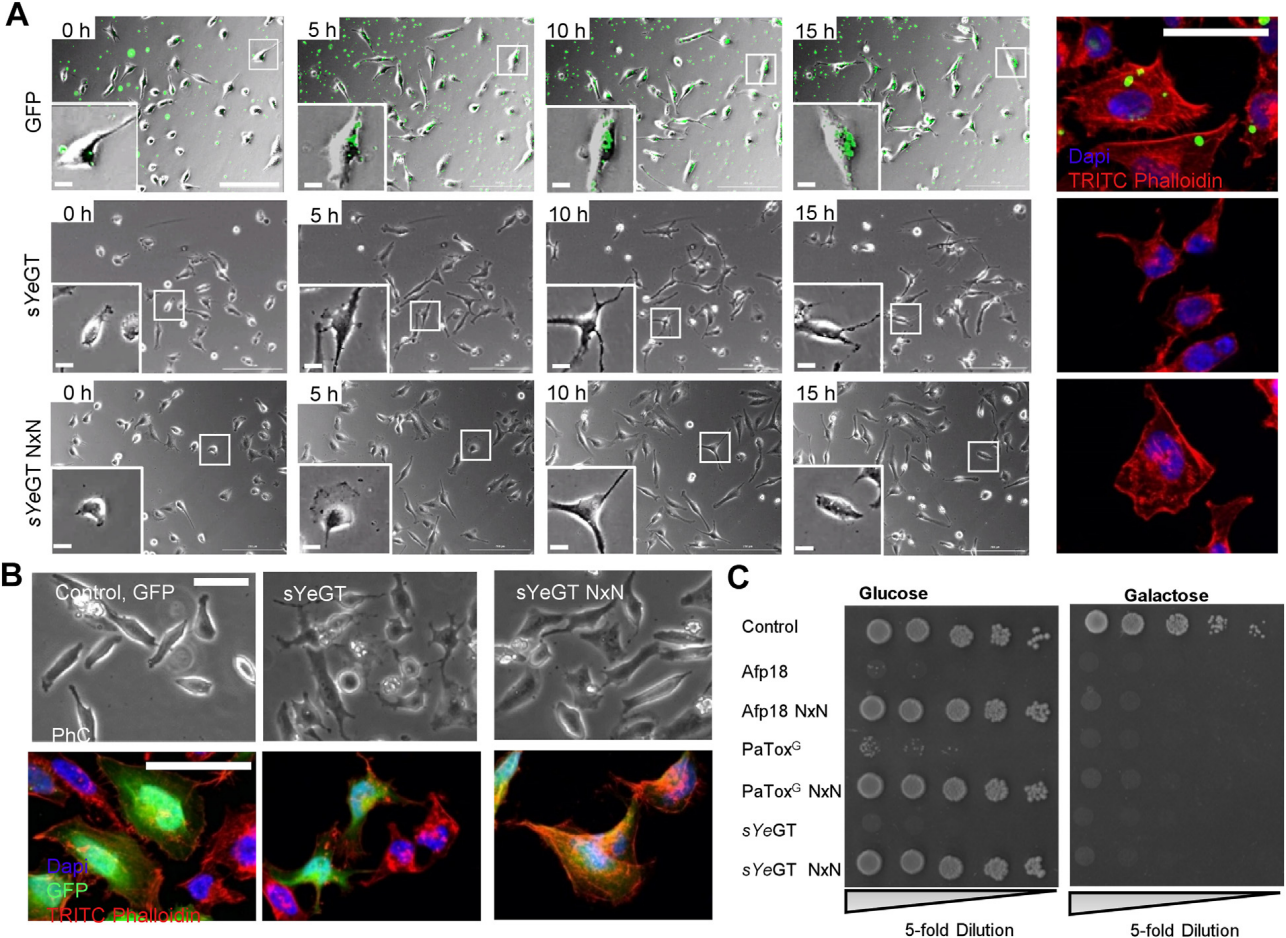
**Morphological changes and toxic effects on eukaryotic cells by sYeGT.***A*, phase contrast (*left* panel) and fluorescence (*right* panel) microscopy images of effects of sYeGT on the actin cytoskeleton. HeLa cells were treated with sYeGT or inactive sYeGT NxN with PROTEOfectene as a delivery system. GFP was used as control for delivery. Actin was stained with TRITC-phalloidin and nuclei with DAPI (4′,6-diamidino- 2-phenylindole). The scale bar represents PhC 200 μm (magnification 20 μm), fluorescence 50 μm. *B*, cellular expression of eGFP coupled sYeGT and inactive sYeGT NxN in HeLa cells transfected *via* Lipofectamine 2000. GFP was used as transfection control. *Top row* shows phase contrast and *bottom row* shows cells with TRITC-phalloidin stained actin and DAPI-stained nuclei. The scale bars represent 50 μm. *C*, drop test analysis of yeast strain *Saccharomyces cerevisiae* MH272α expressing WT or inactive mutant of Afp18, PaTox^G^, and sYeGT under the control of galactose inducible promotor Gal1. The growth phenotype was studied using drop test analysis by 5-fold titration and spotted onto supplemented SD agar (with glucose, Glc) or SGal agar (with galactose, Gal) on SD plates at 30 °C for 3 days. All experiments shown were performed at least three times with similar results. Afp, antifeeding prophage; eGFP, enhanced GFP; TRITC, tetramethylrhodamine isothiocyanate.

Addition of sYeGT to culture medium of cells exhibited no obvious effects. Therefore, we tested various membrane transport methods including protective antigen (PA), the transport unit of anthrax toxin, and PROTEOfectene. While PA was not effective, using PROTEOfectene as transporting agent, recombinant purified sYeGT intoxicated HeLa cells (Fig. 2*A*) and caused time-dependent changes in cell morphology and rearrangement of the actin cytoskeleton. As control, we changed the aspartates of the DxD (Asp150-x-Asp152) motif of sYeGT to asparagine (sYeGT-NxN). This mutant was not able to induce the typical morphological changes of WT sYeGT. Same results were obtained, when GFP-sYeGT constructs were expressed in HeLa cells (Fig. 2*B*). Expression of the WT toxin caused major changes in cell morphology and redistribution of the actin cytoskeleton, while cells expressing the NxN mutant remained unchanged.

Next, we studied the effects of sYeGT and of the inactive NxN mutant in *Saccharomyces cerevisiae* expressed under the control of an inducible galactose promotor (Fig. 2*C*). The growth of yeast transformed with *Yersinia* WT proteins was already decreased using glucose-containing agar, while the NxN mutant exhibited growth. Similar results were obtained with the related glycosyltransferase domain of *P. asymbiotica* toxin PaTox and with the *Y. ruckeri* effector Afp18. After induction of expression by galactose, yeast transformed with WT or mutant sYeGT showed no growth. Same results were obtained with PaTox and Afp18. Thus, even in the presence of glucose, small amounts of active toxins are expressed, which are sufficient to inhibit yeast proliferation. Induction of toxin expression by galactose exhibits a potent effect on yeast even for the mutant protein with a low residual activity.

### Donor- and acceptor substrate specificity of sYeGT

**Figure 3:**
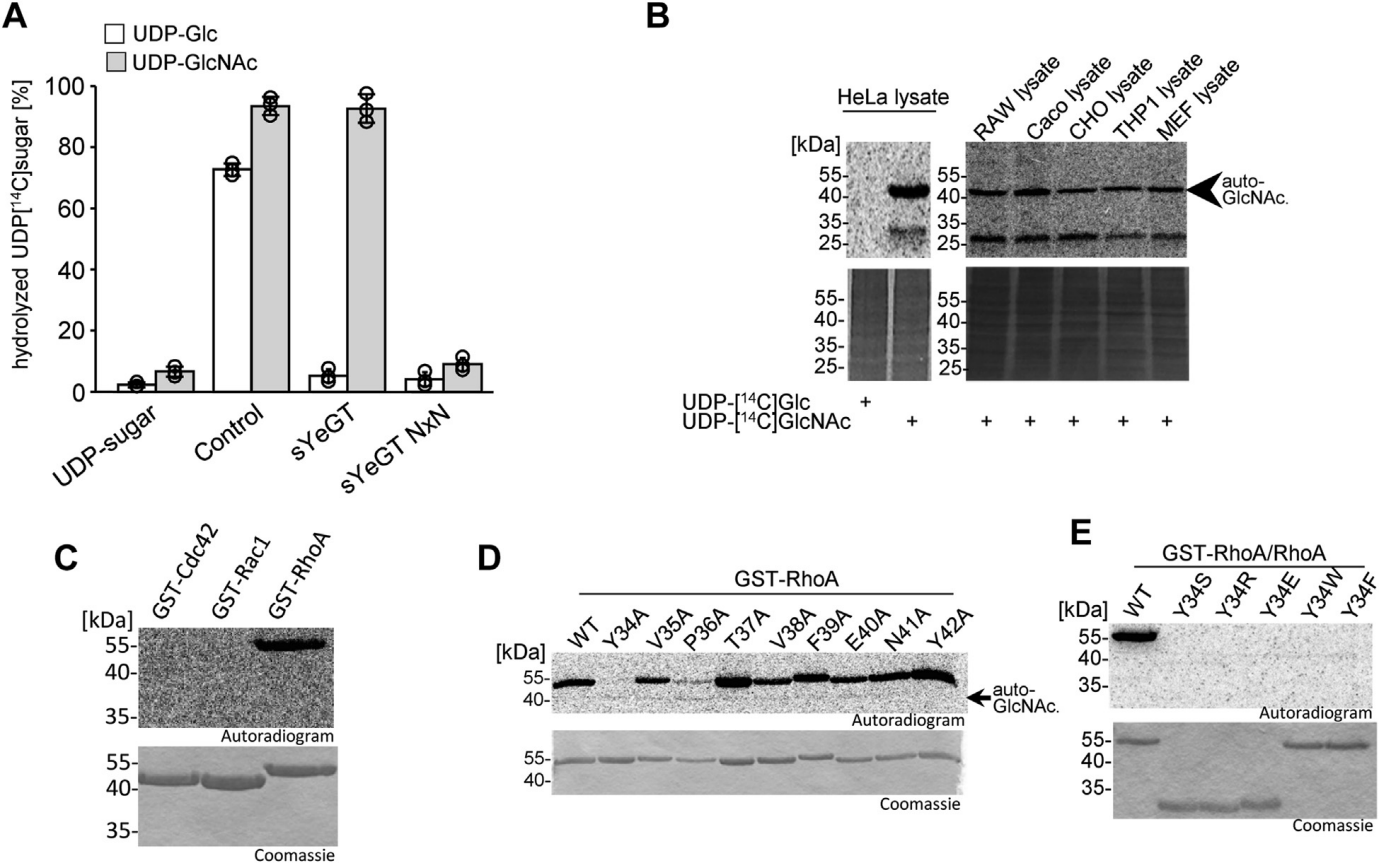
***Yersinia enterocolitica* glycosyltransferase sYeGT GlcNAcylates RhoA at tyrosine 34.***A*, donor substrate specificity of sYeGT determined by glycoside hydrolase activity. Percentage of UDP-[14C]-sugar hydrolyzed was determined by PEI thin layer chromatography and autoradiography after incubation of sYeGT or sYeGT NxN for 0.5 h at 30 °C in 50 mM Hepes, pH 7.4, 2 mM MgCl_2_, and 1 mM MnCl_2_ with 10 μM UDP-[^14^C]Glc (*white bar*) and 10 μM UDP-[^14^C]GlcNAc (*gray bar*). PaTox^G^ was used as positive control for UDP-[^14^C]GlcNAc hydrolysis and TcdB as positive control for UDP-[^14^C]Glc hydrolysis. Error bars indicate SD of three technical replicates. *B*, autoradiography of the SDS–PAGE from different cell lysates (RAW, murine macrophage cells; Caco, human epithelial colon adeno carcinoma cells; CHO, Chinese hamster ovary cells; THP1, human monocyte leukemia cells; and MEF, murine embryonal fibroblasts; each 50 μg) incubated with sYeGT (1 μM) for 0.5 h at 30 °C and indicated radiolabeled UDP-sugars. *C*, autoradiogram and Coomassie-staining to identify substrate *via* GlcNAcylation of small GTPases of the Rho family (each 3 μg of GST proteins) with 100 nM sYeGT and radiolabeled UDP-[^14^C]GlcNAc. Representative autoradiograms and Coomassie stained SDS-Page shown after 0.5 h incubation at 30 °C. *D*, alanine screen for identification of the modification site by *in vitro* GlcNAcylation of GST-RhoA (3 μg) and indicated switch I-region mutants with sYeGT (100 nM) at 30 °C for 0.5 h. Representative autoradiograms (*upper* panel) and Coomassie stained SDS-PAGE (*lower* panel) are shown. A small auto-GlcNAcylation of sYeGT is indicated. *E*, autoradiograms and Coomassie-staining of *in vitro*^14^C-GlcNAcylated GST-RhoA/RhoA (3 μg) and indicated RhoA-Y34 mutants modified by sYeGT (100 nM) at 30 °C for 0.5 h. All experiments shown were performed three times with similar results.

All glycosyltransferase toxins studied so far use UDP-glucose or UDP-GlcNAc as sugar donor. To identify the donor substrate of sYeGT, we performed glycoside hydrolase assays and used UDP-[^14^C]GlcNAc and UDP-[^14^C]glucose sugar donors. These studies revealed that sYeGT preferentially hydrolyzed UDP-[^14^C]GlcNAc (Fig. 3*A*). Subsequently, we used radioactively labeled UDP-[^14^C]GlcNAc for *in vitro* glycosylation of different cell lysates. Thereby, a ∼25 kDa protein was labeled in all tested cell lysates (Fig. 3*B*). In addition, a strong autoglycosylation of sYeGT was observed. By contrast, with UDP-[^14^C]glucose no modification of proteins and no autoglycosylation was detected. Because sYeGT modified proteins with the same mass as Rho GTPases, which are substrate of Afp18 and PaTox, we tested various Rho GTPases in *in vitro* glycosylation assays with sYeGT (Figs. 3*C* and 5*B*). This study revealed that sYeGT modified RhoA, B and C but not the other Rho subfamily proteins. Therefore, we concluded that the preferred acceptor substrates of sYeGT are RhoA, B and C. For the identification of the acceptor amino acid of RhoA, an alanine screen was performed (Fig. 3*D*).

**Figure 4:**
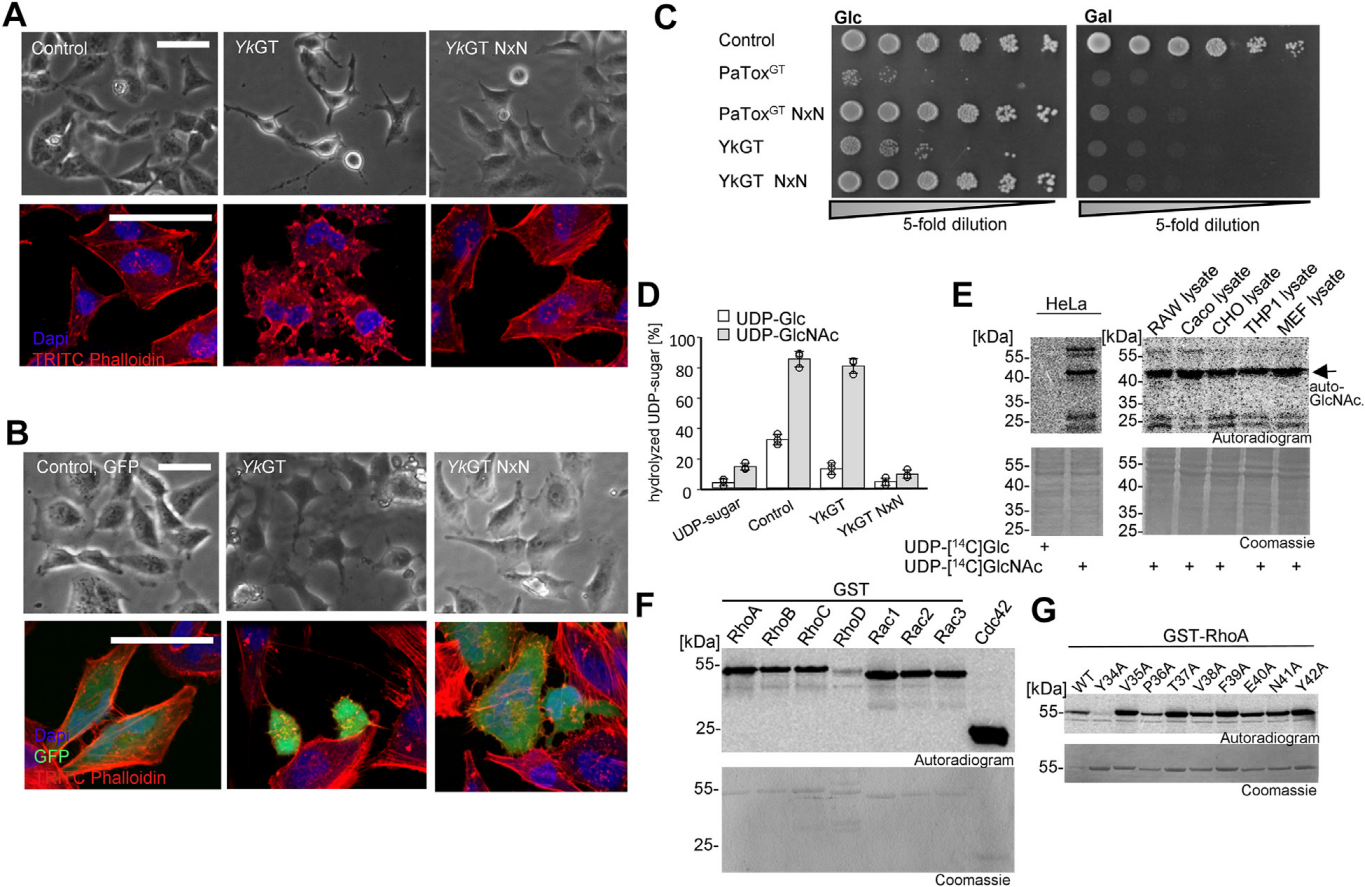
**Morphological changes, toxic effects, and substrate specificity of YkGT.***A*, phase contrast (*upper* panel) and fluorescence (*lower* panel) microscopy images showing the effects of YkGT on the actin cytoskeleton. HeLa cells were treated with YkGT or inactive YkGT NxN using PA (protective antigen from *Bacillus anthracis*) as a delivery system. Actin was stained with TRITC-phalloidin and nuclei with DAPI. The scale bars represent 50 μm. *B*, cellular expression of eGFP coupled YkGT and inactive YkGT NxN in HeLa cells transfected *via* Lipofectamine 2000. GFP was used as transfection control. *Top row* shows phase contrast and *bottom row* shows TRITC-phalloidin-stained actin and DAPI-stained nuclei of cells. The scale bars represent 50 μm. *C*, drop test analysis of yeast strain *Saccharomyces cerevisiae* MH272α expressing WT or inactive mutant of PaTox^G^ and YkGT under the control of the galactose inducible promotor Gal1. The growth phenotype was studied using drop test analysis by 5-fold titration and spotted onto supplemented SD agar (with glucose, Glc) or SGal agar (with galactose, Gal) on SD plates at 30 °C for 3 days. *D*, donor substrate specificity of YkGT determined by glycoside hydrolase activity. Percentage of UDP-[^14^C]-sugar was determined by PEI thin layer chromatography and autoradiography after incubation of YkGT (1 μg) for 0.5 h at 30 °C in 50 mM Hepes, pH 7.4, 2 mM MgCl_2_, and 1 mM MnCl_2_ with 10 μM UDP-[^14^C]Glc (*white* bar) and 10 μM UDP-[^14^C]GlcNAc (*gray* bar). PaTox^G^ was used as positive control for UDP-[^14^C]GlcNAc hydrolysis and TcdB as positive control for UDP-[^14^C]Glc hydrolysis. Error bars indicate SD of three technical replicates. *E*, autoradiography of the SDS-PAGE from different cell lysates (Hela-, RAW-, Caco-, THP1-, and MEF cells; each 50 μg) incubated with YkGT (1 μM) for 0.5 h at 30 °C and indicated radiolabeled UDP-sugars. *F*, autoradiogram and Coomassie-staining to identify protein substrates by GlcNAcylation of small GTPases (each 3 μg; Rho and Rac proteins were used as GST-tag proteins, Cdc42 without tag) with YkGT (100 nM) and radiolabeled UDP-[^14^C]GlcNAc. Representative autoradiograms and Coomassie stained SDS-Page are shown. *G*, alanine screen for identification of the modification site *via in vitro* GlcNAcylation of GST-RhoA and indicated Switch I-region mutants (each 3 μg) with YkGT (100 nM) at 30 °C for 0.5 h. Representative autoradiograms (*upper* panel) and Coomassie-stained SDS-PAGE (*lower* panel) are shown. The sample of GST-RhoA (WT) was diluted 1/10 to prevent overexposure. All experiments shown were performed three times with similar results. DAPI, 4′,6-diamidino- 2-phenylindole; TRITC, tetramethylrhodamine isothiocyanate.

**Figure 5:**
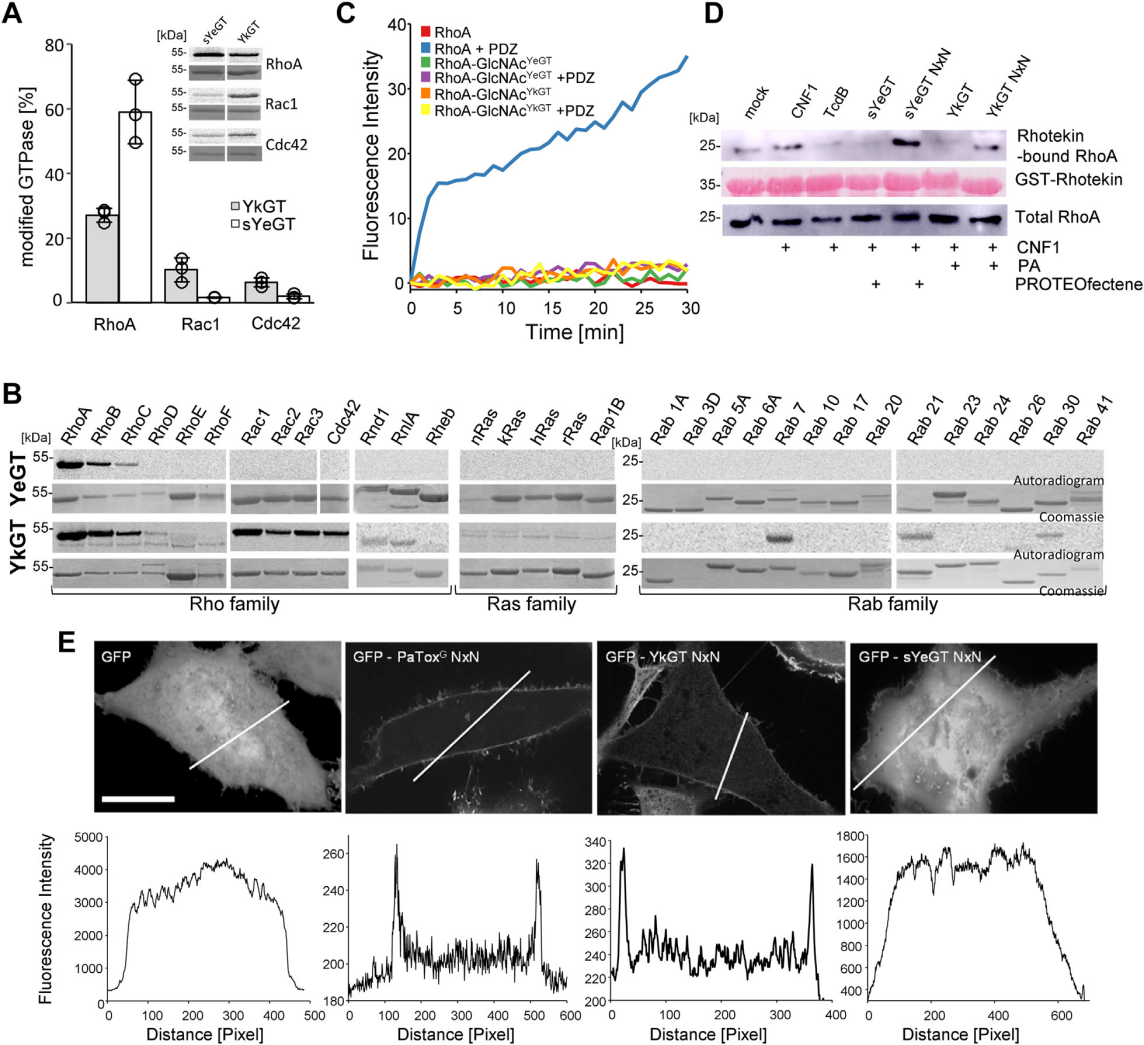
**Consequences of YkGT and sYeGT modification.***A*, quantified modification of GST tagged RhoA, Rac1, and Cdc42 modified by 100 nM sYeGT (*white bars*) and YkGT (*gray bars*) for 0.5 h. Insets show representative autoradiograms (*upper* panel) and Coomassie-stained SDS-PAGE (*lower* panel) after 0.5 h of incubation obtained in individual time courses. Error bars indicate SD of three independent experiments. *B*, autoradiogram and Coomassie staining to identify substrate by acceptor screen *via* GlcNAcylation of 3 μg small GTPases (Rho- and Ras family GTPases were used with and Rab GTPases without GST tag as indicated by different masses) with 100 nM YkGT and sYeGT and radiolabeled UDP-[^14^C]GlcNAc. Representative autoradiograms and Coomassie-stained SDS-PAGE shown after 0.5 h incubation at 30 °C. Experiments were three times repeated with similar results. *C*, nucleotide exchange was measured by mant-GppNHp exchange with GST-RhoA, sYeGT-GlcNAcylated GST-RhoA, and YkGT-GlcNAcylated GST-RhoA in presence and absence of the GEF PDZ. Data are representative for three independent experiments. *D*, Western blot analysis of RhoA pull-down experiments with HeLa cells intoxicated with YkGT (with PA for delivery) and sYeGT (with PROTEOfectene for delivery), the inactive mutants YkGT NxN (with PA for delivery) and sYeGT NxN (with PROTEOfectene for delivery) and (*C*) *difficile* toxin (TcdB). Rho-GTPases were activated with CNF1. Active RhoA was pulled down with Rhotekin-coupled beads. The pulled GTPase was detected by Western blotting using anti-RhoA, antibody. Immunoblot of total RhoA is the loading control. A representative experiment is shown which was three times repeated with similar results. *E*, confocal fluorescence microscopy of HeLa cells expressing eGFP, the eGFP coupled inactive mutants of PaTox^G^, sYeGT, and YkGT. Plasma membrane localization was determined by line scans and quantified *via* densitometry (*bottom*). Data are representative for ≥3 independent experiments. The scale bars represent 15 μm. CNF1, cytotoxic necrotizing factor 1; PA, protective antigen.

We focused on the amino acids of the Switch I-region of RhoA, because the acceptor amino acids of other similar glycosyltransferase toxins like PaTox, Afp18, and TcdB are in this region. The alanine screen revealed that exchange of Tyr-34 to alanine blocked GlcNAcylation. Exchange of Pro-36 to alanine reduced the glycosylation but did not completely block the modification. This indicates that the acceptor amino acid for sYeGT is Tyr-34. In addition, the specificity of the modification site was tested, therefore Tyr-34 was changed to serine, arginine, glutamic acid, tryptophan, and phenylalanine (Fig. 3*E*). sYeGT was only able to modify tyrosine at position 34.

### Effects of YkGT on eukaryotic cells

Next, we studied the putative glycosyltransferase YkGT from *Yersinia kristensenii*. At first, the effect of YkGT was tested in HeLa cells. In this case, the protective antigen PA, the binding and translocation component of anthrax toxin from *Bacillus anthracis* (32) was used to translocate recombinant His-tagged YkGT into the target cell. The filamentous actin was stained with tetramethylrhodamine isothiocyanate (TRITC)-phalloidin after cell fixation (Fig. 4*A*). Again, we used the DxD mutant YkGT-NxN as a control (note, again change of 150Asp-x-152Asp). WT YkGT caused major changes in cell morphology and rearranged the actin cytoskeleton. Cells, which were treated with the inactive YkGT-NxN mutant had the same appearance and morphology as control cells. These results were confirmed by expression experiments. HeLa cells, which expressed WT YkGT, exhibited morphological changes and redistribution of the actin cytoskeleton, while this was not the case after expression of GFP- YkGT-NxN (Fig. 4*B*).

In addition, the effect of YkGT was tested in *S*. *cerevisiae* (Fig. 4*C*) expressed under the control of a galactose-inducible promotor. Similarly, as observed with sYeGT, expression of YkGT but not of the inactive YkGT-NxN mutant largely inhibited yeast growth already in the presence of glucose. After induction of expression by addition of galactose, yeast growth was blocked even with the NxN mutant.

For the identification of the donor substrate, a glycoside hydrolase assay was performed with radioactively labeled UDP-[^14^C]Glc and UDP-[^14^C]GlcNAc (Fig. 4*D*). YkGT cleaved UDP-GlcNAc but not UDP-[^14^C]Glc with the same efficiency as the positive control PaTox, indicating that UDP-GlcNAc is the preferred sugar donor. This result was confirmed by *in vitro* glycosylation of different cell lysates (Fig. 4*E*). While with UDP-[^14^C]Glc no labeling occurred, YkGT modified ∼25 kDa proteins in the presence of UDP-[^14^C]GlcNAc. In addition, an autoglycosylation occurred similarly as observed with sYeGT. In some cell lines, an additional labeling at ∼55 kDa was observed, which was not further studied. Again, similar to *Y. enterocolitica* glycosyltransferase, YkGT modified RhoA, however, in contrast to sYeGT, in addition Rac1, 2, 3, and Cdc42 were modified by the enzyme (Fig. 4*F*). An alanine screen of amino acids from Tyr-34 through Tyr-42 revealed similar results as obtained with sYeGT. Exchange of Tyr-34 strongly inhibited glycosylation of RhoA by YkGT and exchange of Pro-36 reduced the modification. Other amino acid exchanges did not affect the labeling of RhoA. Thus, these data suggest that Tyr-34 of RhoA is the site of YkGT-induced modification (Fig. 4*G*).

### Protein substrate specificity and consequences of GTPase modification by sYeGT and YkGT

To compare the activity and substrate specificity of the two *Yersinia* glycosyltransferases, an *in vitro* glycosylation of RhoA, Rac1, and Cdc42 with 100 nM sYeGT and YkGT was performed and the extend of modification quantified (Fig. 5*A*). The results suggested that both glycosyltransferases prefer RhoA as protein acceptor substrate, however, YkGT additionally modified Rac1 and Cdc42. This finding prompted us to study the substrate specificity in more detail including Rho, Ras, and Rab family proteins (Fig. 5*B*). sYeGT glycosylated only three members of the Rho family (RhoA, B, and C), with RhoA as the preferred substrate and less efficient modification of RhoC. In contrast, YkGT GlcNAcylated RhoA, B, and C and to a minor extend RhoD. Furthermore, Rac1, Rac2, Rac3, and Cdc42 were largely modified. In addition, some Rab proteins were glycosylated including Rab7, Rab21, and Rab30.

Next, we studied the functional consequences of the GlcNAcylation of RhoA. Therefore, we analyzed the influence of glycosylation on the release of RhoA-bound nucleotide induced by the guanine nucleotide exchange factor PDZ (33). At first RhoA was loaded with mant-GppNHp, a nonhydrolyzable GTP analog. The release of mant-GppNHp can be monitored by increase in the fluorescence intensity (Fig. 5*C*). GlcNAcylation of RhoA catalyzed by sYeGT or YkGT blocked the nucleotide exchange induced by PDZ. Additionally, we investigated the interaction of RhoA with its downstream effector Rhotekin in a pull-down assay using sYeGT - or YkGT-intoxicated HeLa cells (Fig. 5*D*). sYeGT was introduced into target cells by using PROTEOfectene as delivery system. For delivery of YkGT into cells, PA was used. To activate Rho GTPases, cells were treated with cytotoxic necrotizing factor 1 (CNF1), which deamidates Rho GTPases and thereby constitutively activates the GTP-binding protein (34). *C. difficile* toxin TcdB was used as a positive control, because it is known to block Rho-effector interaction by glucosylation (17). This experiment showed that both sYeGT and YkGT inhibited the interaction of Rhotekin with RhoA, while the NxN mutants showed no effect.

It has been reported that the intracellular cell membrane localization of glycosylating toxins is essential for their activity on Rho GTPases. This was shown for *P. asymbiotica* toxin PaTox (35) and for *C. difficile* toxin TcdB (4, 36, 37). To study the localization of *Yersinia* glycosyltransferases in target cells, we used the inactive NxN mutants to prevent major morphological changes. After transfection and expression of enhanced GFP (eGFP)-coupled YkGT in HeLa cells, we observed a localization of the enzyme at the plasma membrane by confocal microscopy (Fig. 5*E*). Surprisingly, the shorter glycosyltransferase sYeGT exhibited a distribution in the whole cell similar as the eGFP transfection control.

### Identification of functional amino acids

**Figure 6:**
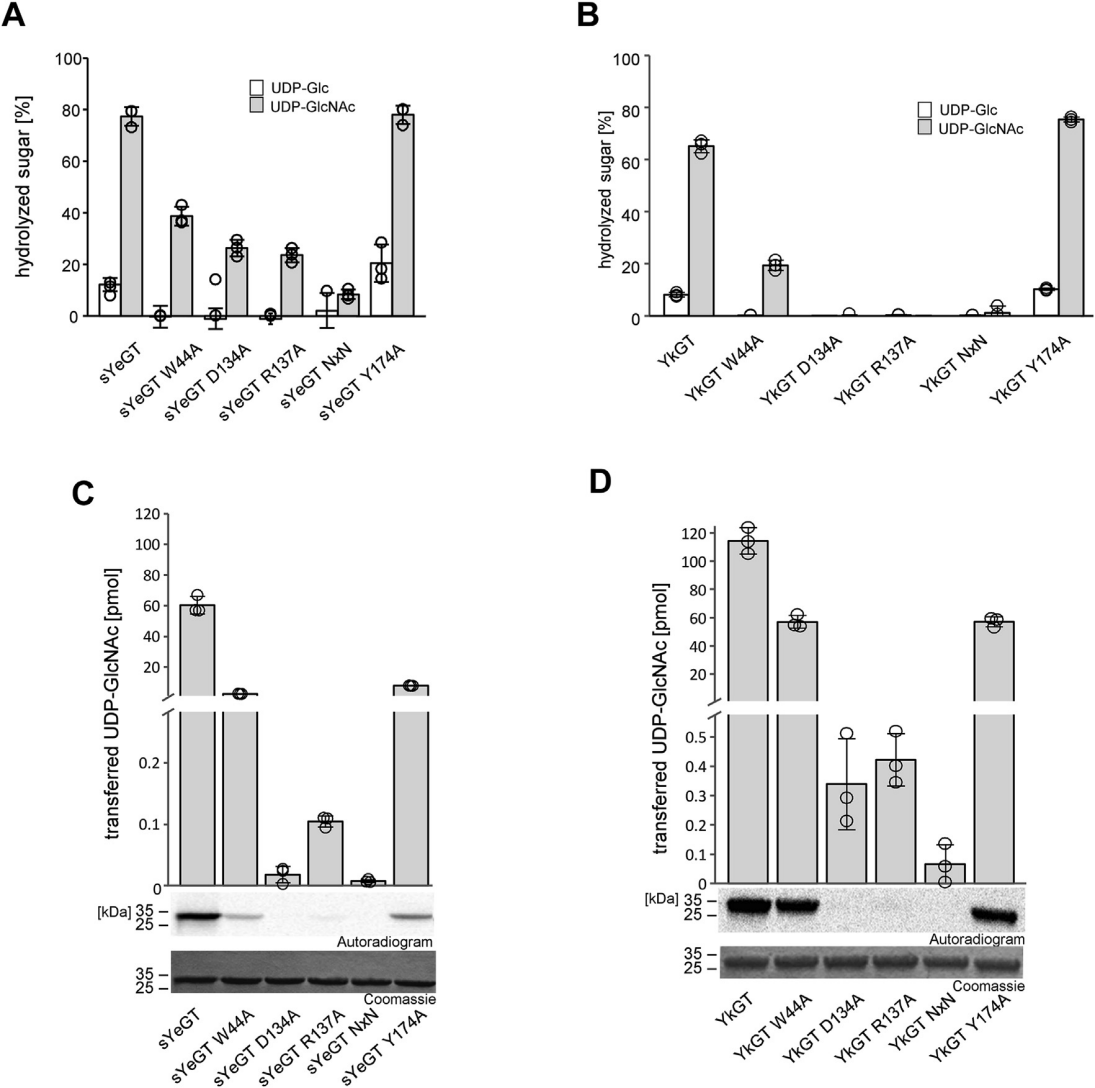
**Identification of functional amino acids in the binding pocket.***A*, influence on the glycoside hydrolase activity of conserved catalytic amino acids in the catalytic pocket of sYeGT. Percentage of UDP-[^14^C]-sugar was determined by PEI thin layer chromatography and autoradiography after incubation of sYeGT (1 μg) for 0.5 h at 30 °C in 50 mM Hepes, pH 7.4, 2 mM MgCl_2_, and 1 mM MnCl_2_ with 10 μM UDP-[^14^C]Glc (*white bar*) and 10 μM UDP-[^14^C]GlcNAc (*gray bar*). Error bars indicate SD of three individual experiments. *B*, influence on the glycoside hydrolase activity of conserved catalytic amino acids in the catalytic pocket of YkGT. Percentage of UDP-[^14^C]-sugar was determined by PEI thin layer chromatography and autoradiography after incubation of sYeGT (1 μg) for 0.5 h at 30 °C in 50 mM Hepes, pH 7.4, 2 mM MgCl_2_, and 1 mM MnCl_2_ with 10 μM UDP-[^14^C]Glc (*white bar*) and 10 μM UDP-[^14^C]GlcNAc (*gray bar*). Error bars indicate SD of three individual experiments. *C*, amount of transferred UDP-[^14^C]GlcNAc in pmol on RhoA (3 μg) by sYeGT mutants (each 100 nM) with changed conserved amino acids in the catalytic pocket. Data are given as means ± SD of three independent experiments. Representative autoradiograms and Coomassie-stained SDS-PAGE are shown after 0.5 h incubation at 30 °C. *D*, amount of transferred UDP-[14C]GlcNAc in pmol on RhoA (3 μg) by YkGT mutants (each 100 nM) with changed conserved amino acids in the catalytic pocket. Data are given as means ± SD of three independent experiments. Representative autoradiograms and Coomassie stained SDS-PAGE are shown after 0.5 h incubation at 30 °C.

To identify residues, which are essential for the glycoside hydrolase and glycosyltransferase activities of sYeGT and YkGT, amino acids from different areas of the proteins were tested in mutagenesis studies. At first, amino acids, which are located in the catalytic pocket of the proteins, were exchanged. The highly conserved tryptophan (W^U^) (Trp-44 in sYeGT and in YkGT), which stabilizes the uracil ring of UDP *via* aromatic stacking, was mutated. Asp-134 and Arg-137 of sYeGT and YkGT, which are part of the sugar binding *GT-A triad* (together with Asp-150 in both proteins), were also changed. In addition, a conserved tyrosine (Tyr-174 in sYeGT and YkGT), located in loop F and suggested to stabilize GlcNAc, was changed (Figs. 6 and S4*A*). The hydrolase assays revealed that the mutations of the DxD motif and the GT-A triad strongly reduce the glycoside hydrolase activity of sYeGT and YkGT. Nevertheless, a residual glycoside hydrolase activity remained in the case of sYeGT mutants, while the respective YkGT mutants were nearly completely blocked. In the glycosylation assay, none of these mutants of the GT-A triad were able to modify the acceptor substrate RhoA to a larger extend (note the different scales of the ordinate). The mutation of the tryptophan (W^U^), which is stabilizing the uracil ring of the UDP residue, decreased the glycoside hydrolase activity of sYeGT only by about 50% and ∼70% in the case of YkGT. A similar small reduction in enzyme activity was also observed in the glycosylation assay. The exchange of the tyrosine Tyr-174 in sYeGT and YkGT, had no influence on the glycoside hydrolase activity. The glycosylation activity of these mutants revealed that the tyrosine is important for the glycosylation, because the activity was reduced by the same extend as by the W^U^ mutant.

**Figure 7:**
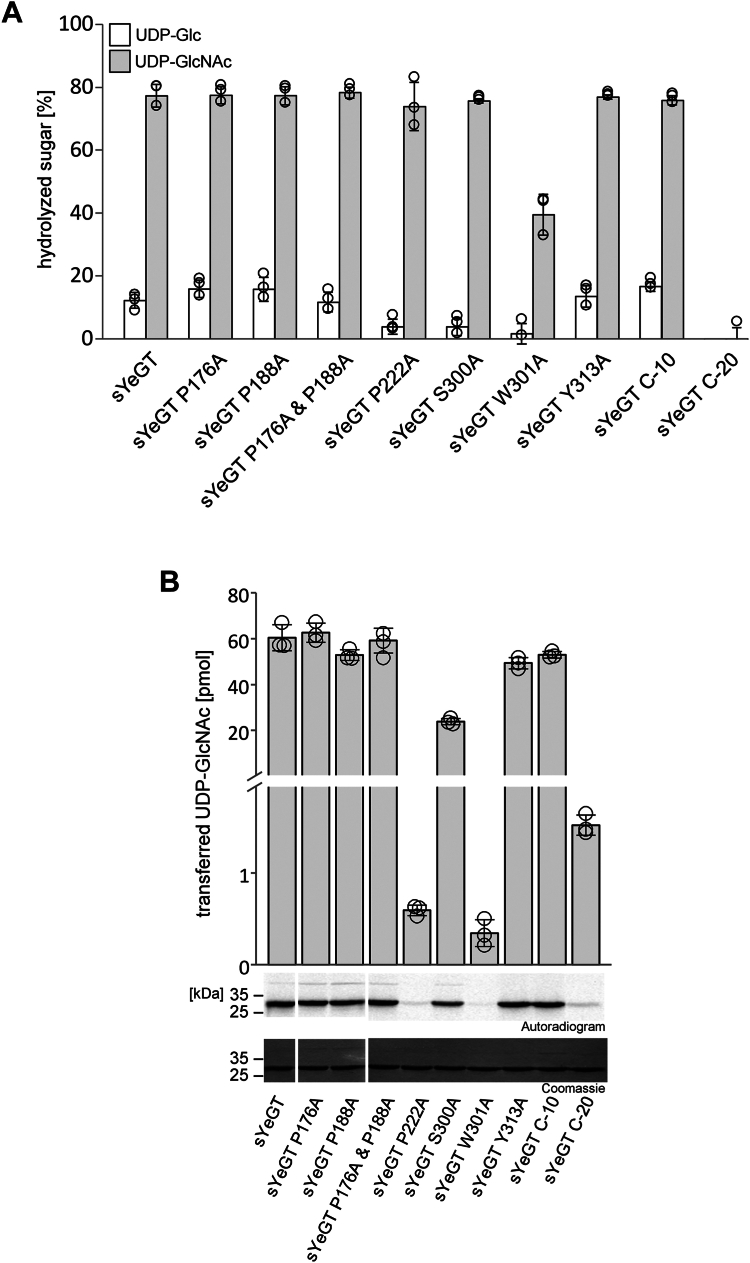
**Identification of amino acids important in glycosyltransferase activity.***A*, influence on the glycoside hydrolase activity of conserved catalytic amino acids in the catalytic pocket of sYeGT. Percentage of UDP-[^14^C]-sugar was determined by PEI thin layer chromatography and autoradiography after incubation of sYeGT (1 μg) for 0.5 h at 30 °C in 50 mM Hepes, pH 7.4, 2 mM MgCl_2_, and 1 mM MnCl_2_ with 10 μM UDP-[^14^C]Glc (*white bar*) and 10 μM UDP-[^14^C]GlcNAc (*gray bar*). Data are given as means ± SD of three individual experiments. *B*, amount of transferred UDP-[^14^C]GlcNAc in pmol on RhoA (3 μg) by sYeGT (100 nM) mutants with changed amino acids as indicated. Data are given as means ± SD of three individual experiments. Representative autoradiograms and Coomassie-stained SDS-PAGE are shown after 0.5 h incubation at 30 °C. (Left control sYeGT is the same sample as in Fig. 6*C*, because mutants sYeGT P176A and sYeGT P188A are from the same gel.).

The second group of mutants, characterized in Figure 7 and in Fig. S4*B*, were derived from the sYeGT crystal structure analysis. We changed Pro-176, Pro-188, and Pro-222, which were assumed to be possibly crucial for the binding pocket folding as they are found in, and next to loop F. Additionally, Ser-300, Trp-301, and Tyr-313 (not visible in structure), which are all located in the flexible C-terminal loop and possibly able to interact with the bound donor, were changed. Moreover, by truncating sYeGT of the 10 C-terminal residues, the tip of this flexible loop was removed. These ten residues are not modeled in the structure due to absence of electron density. Further truncation of 20 C-terminal amino acids removed the complete flexible loop including Ser-300 and Trp-301 (Fig. 7). The glycoside hydrolase activity assay in the presence of these sYeGT mutants and with UDP-GlcNAc as donor revealed that only the W301A mutation located in the flexible loop affected hydrolase activity. Changes in the ability to hydrolyze UDP-Glc were marginal. The glycosylation of RhoA with UDP-GlcNAc was strongly reduced when Pro-222 or Trp-301 were exchanged. In agreement with the importance of Trp-301 for glucosylation, the C-terminal -20 truncation resulted in strongly reduced transferase activity, while deletion of the C-terminal ten amino acids showed no effect.

**Figure 8:**
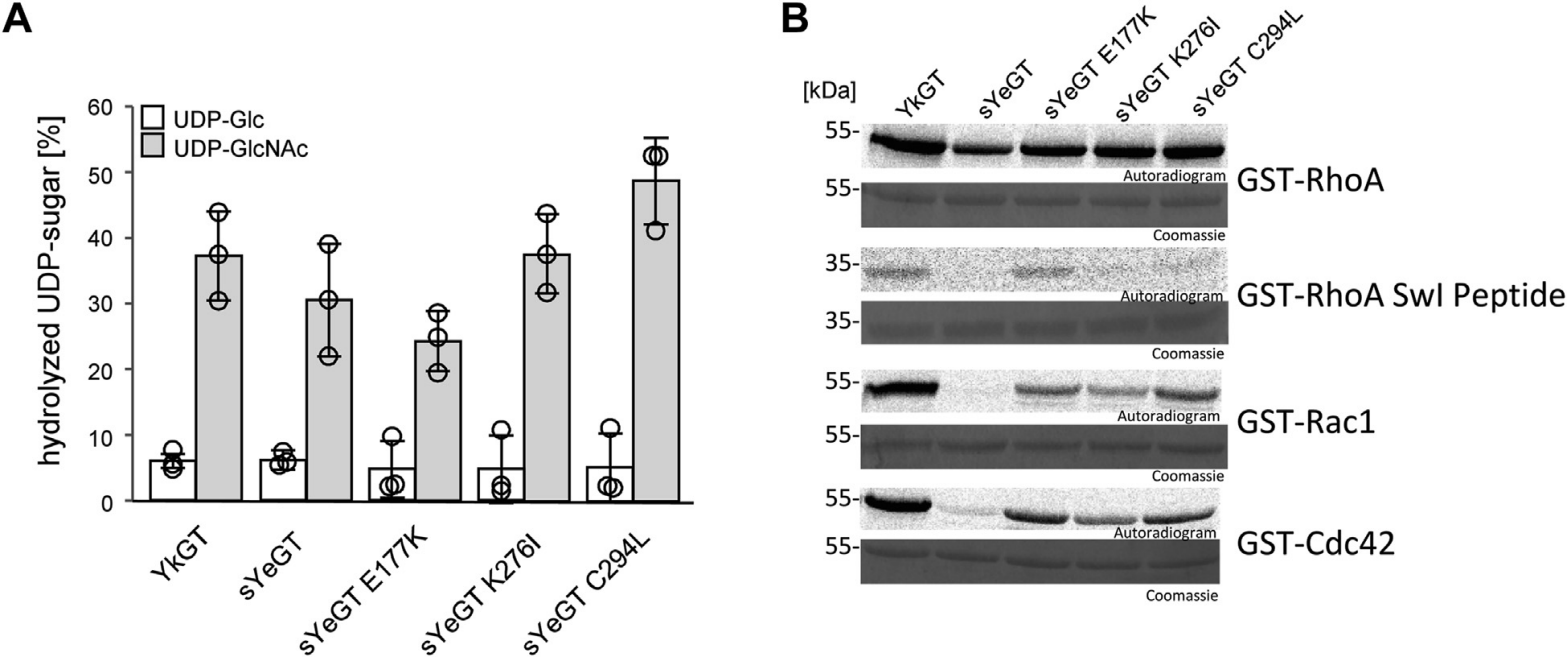
**Change of substrate specificity of sYeGT.***A*, determination of donor substrate specificity by calculating the amount of hydrolyzed UDP-[^14^C]Glc (*white bar*) and UDP-[^14^C]GlcNAc (*gray bar*) by 100 nM YeGT and proteins with mutation on surface amino acids sYeGT E177K, sYeGT K276I, and sYeGT C294L. Data are given as means ± SD of three individual experiments. Representative autoradiograms and Coomassie-stained SDS-PAGE shown after 0.5 h incubation at 30 °C. *B*, autoradiogram and Coomassie staining to test the ability to modify 3 μg RhoA, RhoA Switch I peptide (S25 through V48), Rac1 and Cdc42 by 100 nM YkGT, sYeGT and the sYeGT mutants sYeGT E177K, sYeGT K276I, and sYeGT C294L with UDP-[^14^C]GlcNAc. Representative autoradiograms and Coomassie stained SDS-PAGE shown after 0.5 h incubation at 30 °C. Experiments were repeated three times with similar results.

sYeGT and YkGT are highly similar. In addition to an extended N terminus of YkGT, both enzymes differ only in 12 amino acids. Thus, the fact that YkGT modifies an increased number of substrate proteins as compared to sYeGT is remarkable. Therefore, we changed three amino acids (Glu-177, Lys-276, and Cys-294) in sYeGT, which are surface exposed and near the catalytic pocket or on the front side of the protein, to the residues found in YkGT (Fig. S4*C*). Glu-177 that is located at the tip of loop F was changed to lysine. Lys-276 which is located at the front side of the 3-helix bundle was changed to isoleucine. Finally, Cys-294, located at the base of the flexible C-terminal loop, was changed to leucine. Glycoside hydrolase activities and protein substrate specificity of all the mutants were tested and compared to WT sYeGT and YkGT (Fig. 8, *A* and *B*). Notably, the glycoside hydrolase activities of all mutants were similar to the WT proteins (Fig. 8*A*). While WT sYeGT modified RhoA only, all sYeGT mutants (E177K, K276I, and C294L) were able to modify RhoA, Rac1, and Cdc42 (Fig. 8*B*). However, the extent of modification was in each case lower than the respective modifications by YkGT. Interestingly, sYeGT E177K like YkGT was able to modify a RhoA peptide, covering 22 amino acids of switch I-domain.

### Truncation and extension of sYeGT and YkGT

**Figure 9:**
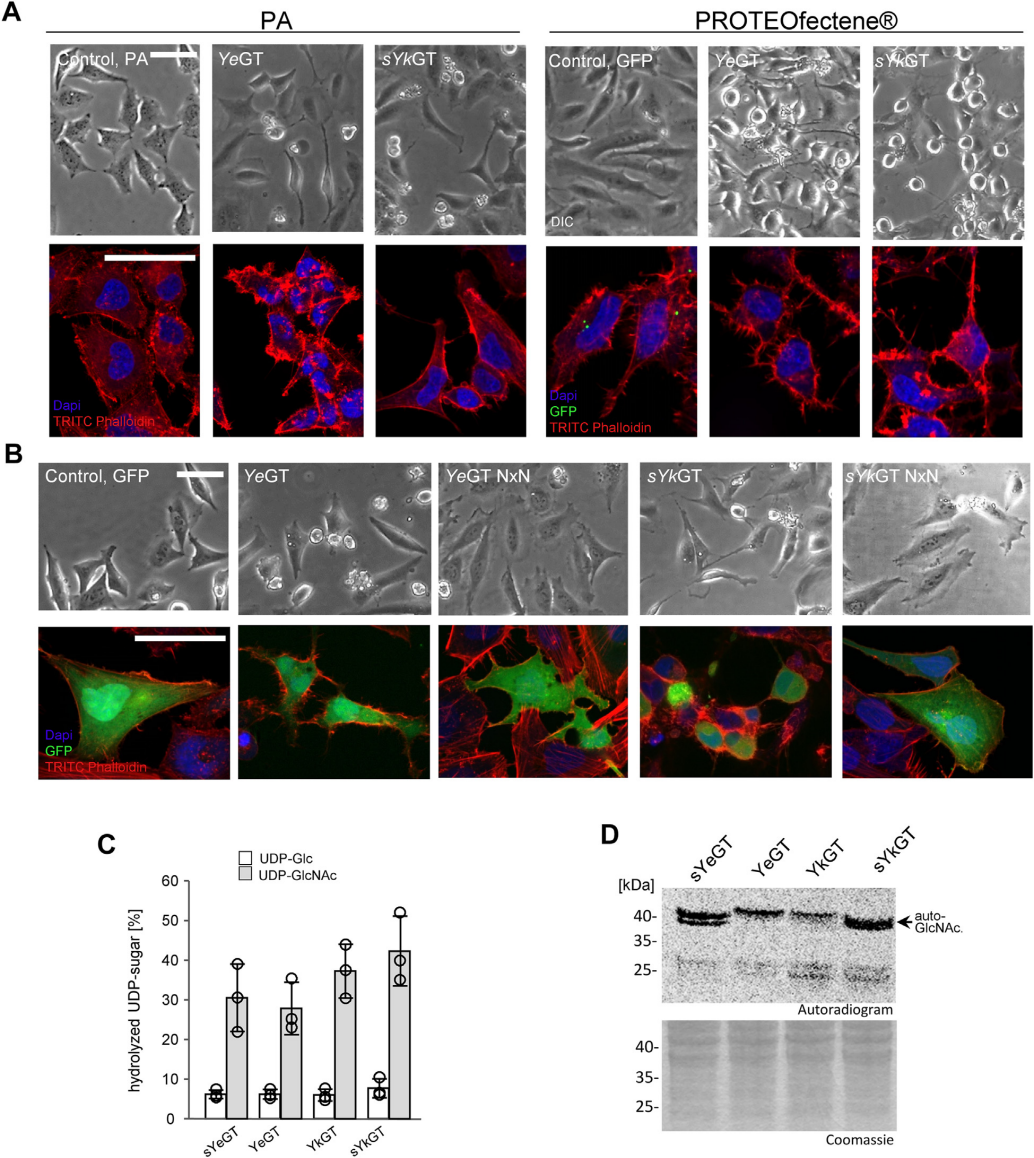
**Role of the N-terminal amino acids sequence of YeGT and YkGT.***A*, phase contrast (*upper* panel) and fluorescence (*lower* panel) microscopy images of cell intoxication, using PA and PROTEOfectene as transporting agent, show the effects of full length YeGT and sYkGT (deletion of the 16 N-terminal amino acids) on the actin cytoskeleton. GFP was used as control for delivery with PROTEOfectene. Actin was stained with TRITC-phalloidin and nuclei with DAPI. The scale bar represents 50 μm. Experiments were repeated three times with similar results. *B*, cellular expression of eGFP-coupled YeGT and sYkGT in HeLa cells transfected *via* Lipofectamine 2000. GFP was used as transfection control. *Top row* shows phase contrast and *bottom row* shows actin TRITC-phalloidin-stained actin and DAPI-stained nuclei of cells. The scale bars represent 50 μm. Experiments were repeated three times with similar results. *C*, donor substrate specificity of YeGT and sYkGT in comparison to sYeGT and YkGT was determined by glycoside hydrolase activity. Percentage of UDP-[^14^C]-sugar was determined by PEI thin layer chromatography and autoradiography after incubation of 1 μg of *Yersinia* proteins (as indicated) for 0.5 h at 30 °C in 50 mM Hepes, pH 7.4, 2 mM MgCl_2_, and 1 mM MnCl_2_ with 10 μM UDP-[^14^C]Glc (*white bar*) and 10 μM UDP-[^14^C]GlcNAc (*gray bar*). Error bars indicate SD of three independent experiments. *D*, autoradiography of the SDS-PAGE from HeLa cell lysates (50 μg) incubated with sYeGT, YeGT, YkGT, and sYkGT (each 1 μM) for 0.5 h at 30 °C. Experiment was repeated three times with similar results. PA, protective antigen; TRITC, tetramethylrhodamine isothiocyanate.

As shown above, main parts of our study were performed with a short version of YeGT (sYeGT), which allowed crystal structure analysis of the protein. Therefore, we wanted to know which functional differences are observed with the long version of the protein (YeGT). Moreover, for comparison, we studied a short version of YkGT (sYkGT), which is N terminally deleted of 16 amino acids. These two additional versions, YeGT and sYkGT, were constructed and recombinantly expressed in *Escherichia coli*. With both constructs, HeLa cells were intoxicated using PA and PROTEOfectene as transporting agents (Fig. 9*A*). Intoxication using PA caused morphological changes of cells, which were incubated with the long version of YeGT. Cells, incubated with the truncated sYkGT construct, behaved like controls without toxin. When PROTEOfectene was used as transporting agent, the cells incubated with both constructs changed their morphology and exhibited rearrangement of the actin cytoskeleton, resulted in cell rounding. Thus, efficient PA-induced translocation of the *Yersinia* protein was possible only with the 16 amino acids at the N terminus. In transfection experiments, the effects of the extended version YeGT and the truncated sYkGT on HeLa cells were tested with eGFP fusion constructs (Fig. 9*B*). Cells expressing the long version of YeGT or the truncated sYkGT construct changed their morphology, and the actin cytoskeleton was rearranged. Cells expressing the inactive NxN mutants showed no cell rounding.

Next, we tested whether the N-terminal amino acids influence the enzyme activity of YeGT and YkGT (Fig. 9*C*). These studies revealed no change in donor substrate specificity. The glycoside hydrolase activity of the extended YeGT construct (sYeGT+16aa) was slightly reduced (not significant) a minimal increase (not significant) in activity was observed with the truncated sYkGT construct (YkGT-16aa) (Fig. 9*C*). In HeLa lysate (Fig. 9*D*), all transferases studied showed autoglycosylation, which appeared to be stronger with the shorter protein versions. Moreover, a modification of an ∼25 kDa substrate could be detected. Thus, the N-terminal 16 amino acids seem to be important for the translocation *via* PA, but not for enzyme activity.

## Discussion

In this study, we identified and characterized two glucosylating toxins/effectors, which are harbored by specific strains of *Y*. *enterocolitica* and *Y*. *kristensenii*. The amino acid sequences of these two glycosyltransferases are 96% identical and highly similar to the GTPase GlcNAcylating effector domains of PaTox and Afp18 produced by *P*. *asymbiotica* and *Y*. *ruckeri*, respectively. Determination of the high-resolution crystal structure of the *Y. enterocolitica* protein revealed high structural similarities to the glycosyltransferase domain of PaTox (PaTox^G^).

In spite of the high sequence similarity between sYeGT and YkGT, both enzymes differ in their substrate protein specificity. While sYeGT, like Afp18, modifies RhoA, B, and C, YkGT is able to glycosylate a broader spectrum of acceptor substrates also including Rac1, Rac2, Rac3, Cdc42, and Rab7. Both enzymes modify Tyr-34 of RhoA. Tyr-34 is located in the conserved switch I-domain of small GTPases (38). This domain regulates the interaction with GTPase-activating proteins, GEFs, and effectors (16, 26).

Intoxication and expression experiments with sYeGT and YkGT in HeLa cells revealed changes in cell morphology, which are typical for inactivation of Rho proteins (39). Interestingly, sYeGT was not able to enter the cells when *B*. *anthracis* PA was used as transporting agent. This problem was circumvented by using the proteofection reagent PROTEOfectene. Also surprising was the finding that sYeGT does not localize to the cell membrane in host cells like PaTox^G^ (35) and YkGT.

The crystal structure of sYeGT revealed a compact protein with a GT-A structural fold. The catalytic pocket of sYeGT is highly conserved in all GT-A glycosyltransferases and harbors the typical DxD motif involved in coordination of the divalent metal ion required for nucleotide coordination (24, 40). The DxD motif of YeGT and YkGT shows a special characteristic, which is the extension to DxDD like in PaTox^G^ and Afp18 (16, 26). The third aspartic acid is also coordinating the divalent metal ion, and thus UDP, *via* a water-mediated hydrogen bond. Considering the relatively small size of YeGT, YkGT, PaTox^G^, and Afp18 compared to the larger clostridial glycosyltransferase toxins, this group of smaller enzymes could be considered as a subfamily. Most likely all these small glycosyltransferases might be placed in the same GT-family as PaTox^G^, which belongs at present to the retaining GT32 family of the CAZy database system.

In the sYeGT structures, the C-terminal flexible loop, also known as lid domain, is partially resolved. It adopts two conformations, depending if the protein has a nucleotide bound or not. We also identified another loop (loop F, residues 171–188) that is changing conformation upon binding of the nucleotide and might help enclosing the sugar moiety of the substrate.

Functionally important amino acids of the *Yersinia* transferases were identified. The mutation of the tryptophan (W^U^) residue of the WIG motif, which is forming a π-stacking interaction with the uracil moiety of the activated sugar donor, decreased the glycoside hydrolase and glucosyltransferase activities but did not completely block the activities. In contrast, mutation of Asp-134 and Arg-137, which are part of the GT-A triad, strongly reduced the transferase activities.

The mutation of Tyr-174, located in the loop F of sYeGT, had no influence on the glycoside hydrolase activity; however, it reduced the glycosylation of Rho proteins, suggesting an essential role in the transferase reaction.

Mutants of sYeGT were constructed to get insights into the functions of various proline residues (Pro-176, Pro-188, and Pro-222) located near the active site or participating in the folding of loop F. The glycosylation of RhoA was strongly reduced with P222A. However, this mutant exhibited the same glycoside hydrolase activity as WT sYeGT suggesting that the transferase reaction is especially affected by this mutation. By contrast, exchange of Trp-301 of the proposed flexible C-terminal loop resulted in reduction of both glycoside hydrolase and glycosyltransferase activities, indicating a role in enzyme activity even without an acceptor protein substrate.

A main difference in the properties of sYeGT and YkGT is the extended acceptor substrate spectrum of the latter enzyme. In order to investigate the effect of single amino acids on the selectivity of acceptor substrates, we changed amino acids Glu-177, Lys-276, and Cys-294 in sYeGT to that of YkGT. Exchange of Glu-177, to lysine was most effective to gain a broader substrate specificity of sYeGT. Change of the charge might be the reason for increased glycosylation of Rac and Cdc42.

In the course of the inspection of published sequenced genomes of *Y*. *enterocolitica* and *Y*. *kristensenii*, it became clear that in addition of a short version of YeGT (sYeGT), which allowed proper crystal structure analysis, a long version exists, which was subsequently compared with the short toxin version. Vice versa, a short version of YkGT was compared with the full-length transferase. In intoxication experiments, the extended YeGT was able to intoxicate HeLa cells *via* PA, while the truncated sYkGT was without effects under these conditions. Both transferases were toxic for cells, when they were transferred into target cells *via* PROTEOfectene and in expression experiments. Thus, the proteins are able to translocate into a host cell *via* PA only when the basic N-terminal 16 amino acids (pI ∼ 10) are present. Previous studies by Collier *et al.* showed that N-terminal basic residues increase transport *via* PA (41, 42).

What are the functional roles of both *Yersinia* enzymes? Deduced from their activity in mammalian cells and their characterization as Rho protein-inhibiting agents, they are most likely bacterial effectors. Probably, they are no toxins (exotoxins) in the strict sense, because bacterial toxins are commonly characterized by their ability to intoxicate cells in the absence of the producing bacteria. Both *Yersinia* enzymes are apparently not able to enter target cells by themselves. At present, the translocation mechanism by which these effectors reach the cytosol of target cells is not known. Afp18, the tyrosine-GlcNAcylating effector from *Y. ruckeri*, is part of the Afp translocation system (28), which is related to type VI secretion systems. We were not able to identify possible transport systems in the vicinity of the genes encoding both enzymes in the respective bacteria. Thus, important questions of future studies are how these enzymes are translocated into target cells.

## Experimental procedures

### Materials, bacterial strains, and plasmids

DNA-modifying enzymes were from Fermentas, Phusion High-Fidelity DNA Polymerase from New England Biolabs, and PfuUltra HF DNA Polymerase from Stratagene. UDP-[^14^C]glucose and UDP-[^14^C]N-acetylglucosamine were from Biotrend. Mant-guanosine 5′-[β,γ-imido]triphosphate (mant-GppNHp) was from Jena Bioscience. The pET28a vector was from Novagen, pGEX-4T1 and pGEX-4T3 were from GE HealthCare. The gene was synthesized as gBlock Gene Fragments from Integrated DNA Technologies, Inc. *Escherichia. coli* TG1 were used for general cloning and protein expression of pGEX constructs. *E. coli* BL21 (DE3) CodonPlus (Stratagene) were used for protein expression of pET constructs. The plasmids pGEX4T1-PDZ-RhoGEF (residues 712–1081) were kindly provided by M. Reza Ahmadian (University of Düsseldorf). Yeast strain *S. cerevisiae* MH272α was provided from Sabine Rospert (Freiburg, Germany) and vector pK92 was provided from Yury Belyi (Gamaleya Institute, Moscow). All other reagents were of analytical grade and purchased from commercial sources.

### Antibodies

Anti-RhoA (26C4, dilution 1:400) antibody was from Santa Cruz, anti-Rac1 (Mab 102, dilution 1:5000) from BD Bioscience, anti-Rac1 (23A8, dilution 1:2500) from Millipore, anti-Cdc42 (17–299, 1:500) from Upstate, and horseradish peroxidase–linked anti-mouse antibody from Rockland Immunochemicals (dilution 1:3000). Antibody specifications are on manufacturer`s website.

### Cloning of genes for bacterial expression

The synthetic DNA fragments were ligated into a predigested pET28a vector with an introduced tobacco etch virus (TEV) protease cleavage site. QuikChange Kit (Stratagene)in combination with PfuUltra HF DNA polymerase was used for the replacement of one to three nucleotides, using the oligonucleotides shown in Table S1. All sequences of corresponding plasmids were confirmed by sequencing (GATC Inc).

### Recombinant-protein expression

*E. coli* BL21∗ CodonPlus cells (Stratagene) transformed with the desired plasmids were grown in LB medium supplemented with the corresponding antibiotics on a shaker at 37 °C until *A*_600_ = 0.8. Protein expression from the pET28-based plasmids was induced by 1 mM IPTG (Roth) for 4 to 5 h at 22 °C, and pGEX-based expression was induced with 0.2 mM IPTG at 37 °C for 6 h. Bacterial cells were harvested by centrifugation at 6,000*g* for 15 min, resuspended in lysis buffer (50 mM Tris–HCl, pH 7.4, 150 mM NaCl, 25 mM imidazole, 30 μg/ml DNase I, 1 mM β-mercaptoethanol, and protease-inhibitor cocktail COMPLETE (Roche)) and lysed by ultrasonic treatment. The cleared lysate was subjected to chromatography on a glutathione-Sepharose or nickel-equilibrated chelating Sepharose Fast Flow column (GE HealthCare) according to the manufacturer’s instructions. Bound proteins were eluted with 10 mM reduced glutathione or 0.5 M imidazole, thrombin treatment, or TEV protease treatment, depending on the construct used. Thrombin was removed by binding to benzamidine-Sepharose (GE HealthCare) and His-TEV by Ni^2+^-affinity chromatography. Further purification was achieved by size-exclusion chromatography with Superdex 75 or Superdex 200 (each 10/300) columns. For crystallization, YeGT, sYeGT, and YkGT were expressed and purified as His-tagged protein with the method of Studier (43). After removal of the His tag by TEV-protease treatment, the proteins were additionally purified by cation-exchange chromatography (Resource S, GE HealthCare) and size-exclusion chromatography (Superdex 75, GE HealthCare).

### Cell culture

HeLa (ATCC CCL-2) and RAW 264.7 (ATCC TIB-71) cells were cultured in Dulbecco’s modified Eagle’s medium supplemented with 10% fetal calf serum, 1% nonessential amino acids, 4 mM penicillin, 4 mM streptomycin, and 1% sodium pyruvate (Biochrom). Cells were cultivated in a humidified atmosphere of 5% CO_2_ at 37 °C. For intoxication and transfection of cells, cells were starved overnight in Dulbecco’s modified Eagle’s medium without fetal calf serum, GST-CNF1 (400 ng/ml), native full-length *C. difficile* toxin TcdB (1 ng/ml) were applied into the medium or intoxicated with His-sYeGT *via* PROTEOfectene (Biontex) according to the manufacturer’s instructions, His-YkGT (20 nM) were applied into the medium in a combination of *B. anthracis* (20 nM) and incubated for 18 h at 37 °C and incubated overnight if not otherwise stated. Cell transfection was performed using Lipofectamine 2000 (Invitrogen) for 2 days according to the manufacturers’ instructions.

### Actin staining

HeLa cells grown on 8-well μ-slide from ibidi GmbH were washed with PBS, fixed with 4% formaldehyde in PBS and permeabilized with 0.15% (v/v) Triton X-100 in PBS for 10 min at room temperature. Subsequently, the cells were incubated with TRITC-conjugated phalloidin and DAPI (1,4-diazobicyclo[2.2.2]octane; Sigma-Aldrich), washed again with PBS. Cells were kept on PBS and analyzed by fluorescence microscopy with an Axiovert 200M microscope (Carl Zeiss) with plan-apochromat objectives, a Yokogawa CSU-X1 spinning disk confocal head with emission filter wheel, and a Coolsnap HQ II digital camera with 405-, 488-, and 561-nm laser lines driven by VisiView (Visitron). All images were processed with Metamorph software (https://www.metamorph.de/).

### Yeast growth assay

To analyze the toxic effects on yeast growth, *S. cerevisiae* cells expressing sYeGT and YkGT (44) and the corresponding mutants were titrated 5-fold from the starting value of *A*_600_ = 1.0. From each dilution, an aliquot of 4 μl of suspension was dropped onto SD agar, supplemented with the corresponding marker substances. Petri plates were incubated for 3 to 5 days at 30 °C (or at the temperature mentioned in figure legends) before photography.

### Hydrolysis of UDP sugars by YeGT

UDP-sugar hydrolysis was measured as described earlier for the glycosyltransferase toxin B (TcdB) from *C. difficile* (45). sYeGT (100 nM) was incubated with 10 μM UDP-[^14^C]sugars in a buffer containing 50 mM Hepes, pH 7.5, 2 mM MgCl_2_ and 1 mM MnCl_2_. Total volume was 10 μl. After incubation for indicated time intervals at 30 °C, samples of 1 μl were taken and subjected to PEI-cellulose thin-layer chromatography (Merck) with 0.2 mM LiCl as mobile phase to separate the hydrolyzed sugar from intact UDP sugar. The plates were dried and analyzed by Phosphorimager. Quantification was carried out with ImageQuant (https://www.cytivalifesciences.com/en/us/shop/protein-analysis/molecular-imaging-for-proteins/imaging-software/imagequant-tl-10-2-analysis-software-p-28619).

### Glycosylation reaction

Recombinant sYeGT proteins (100 nM or 1 μM) were incubated with 10 μM UDP-[^14^C]*N*-acetylglucosamine or UDP-[^14^C]glucose, respectively, indicated lysates (50 μg) or recombinant purified GTPases (2 μM or 3 μM) in a buffer, containing 50 mM Hepes, pH 7.4, 2 mM MgCl_2_, and 1 mM MnCl_2_ for indicated time points at 30 °C. Total volume was 20 μl. Labeled proteins were analyzed by SDS-PAGE and phosphorimaging. All glycosylation reactions were repeated three times.

### Measurements of nucleotide exchange reactions

Preglycosylated RhoA (0.5 μM) and WT RhoA (0.5 μM) were incubated with mant-GppNHp (1 μM) in degassed buffer C (10 mM Tris, pH 7.5, 150 mM NaCl, and 2.5 mM MgCl_2_) at 30 °C. After 30 min, GEFs leukemia-associated PDZ-RhoGEF was added to a final concentration of 200 nM and incubated for the indicated time periods. Fluorescence of bound mant-GppNHp was measured with an LS50B spectrofluorometer at λ_em_ = 460 nm (λ_ex_ = 360 nm). Each experiment was repeated three times.

### Effector pull-down assay

The Rho-binding region of Rhotekin (amino acids 1–90) and the CRIB-domain of PAK (amino acids 56–272) were expressed as GST-fusion protein in *E. coli* BL21 and purified by affinity chromatography with glutathione-Sepharose beads (GE HealthCare). HeLa cells were treated for 15 h with or without GST-CNF1 (400 ng/ml) to activate Rho GTPases and a combination of PA (0.5 mg/ml) and YkGT (100 ng/ml) or *C. difficile* toxin B (TcdB, 1 ng/ml) or sYeGT transported *via* PROTEOfectene (Biontex) according to the manufacturer’s instructions. Cells were lysed in ice-cold GST-Fish buffer (50 mM Tris, pH 7.4, 100 mM NaCl, 1% NP-40, 10% glycerol, 2 mM MgCl_2,_ and 1 mM phenylmethanesulfonylfluoride), and cellular debris was removed by centrifugation (3 min, 17,000 g). A fraction of the cleared lysates (50 μg of total protein) was analyzed by immunoblotting to detect total amounts of the respective GTPases. The lysates were incubated for 1 h at 4 °C with GST-Rhotekin or GST-PAK immobilized on beads. The beads were precipitated and washed with GST-fish buffer. Finally, the proteins were subjected to SDS–PAGE and transferred onto polyvinylidene difluoride membranes and GTPases detected by using specific antibodies and horseradish peroxidase-linked secondary antibodies.

### Crystallization of sYeGT

For crystallization, recombinant proteins were produced by heterologous expression and purified as described above. As the HisTag was removed by treatment with TEV protease, the resulting sYeGT construct, as for the other YeGT and YkGT constructs in this study, comprised an N-terminal Gly-Ser-Met left over after cleavage. The protein in 20 mM Tris pH 7.5, 150 mM NaCl was concentrated to 10 to 40 mg/ml and either directly crystallized or supplemented with 2 mM UDP-*N*-acetylglucosamine (UDP-GlcNAc) and 2 mM MnCl_2_ prior crystallization. The sitting-drop vapor-diffusion method at 20 °C was used and the protein was mixed in a 1:1 ratio with the reservoir solution. Crystals of ligand-free sYeGT appeared within a few days with protein concentrated to 40 mg/ml and reservoir solution containing 10 mM Tri-sodium citrate and 16% PEG 6000. In contrast, crystals of sYeGT supplemented with UDP-GlcNAc and MnCl_2_ appeared only after several weeks in a condition containing 100 mM Hepes pH 7.5, 500 mM sodium acetate, and 50 mM cadmium sulfate. In this case, the protein concentration was 30 mg/ml. Crystals were harvested after cryo-protection using 15 to 20% ethylene-glycol and transferred in liquid nitrogen.

### X-ray diffraction data collection, processing, and refinement

Diffraction data were collected at 100 K on beamline ID23-1 of the European Synchrotron Radiation Facility Data were processed using AutoProc (46) and integrated with aimless (47). The diffraction data showed anisotropy, which was mild for the ligand bound dataset, with only one axis along which the resolution was slightly reduced (resolution limits of 1.30, 1.12, and 1.19 Å along a∗, b∗, and c∗, respectively). In contrast, the ligand-free dataset showed significantly lower resolution along two axes (resolution limits of 2.84, 2.84, and 2.21 Å along a∗, b∗, and c∗, respectively). The anisotropy was analyzed and corrected with STARANISO (48). The diffraction data from the ligand-bound YeGT crystals were initially processed in the orthorhombic space group P2_1_2_1_2_1_. However, careful analysis with Phenix.Xtriage (49) and testing of different space groups showed that the crystals were belonging to space group P2_1_ and were suffering from pseudomerohedral twinning (twin law h, -k, and -l) with a twinning fraction of 0.47. The phase problem was solved by molecular replacement using the program Phaser (50) and the structure of the homologous PaTox glycosyltransferase (Protein Data Bank ID: 4MIX) as search model for the high resolution dataset. This refined structure was used to solve the phase problem of the ligand free data by molecular replacement. Several cycles of model building using COOT (51) and refinement using Phenix.refine (49) or Refmac (52) using TLS were performed to obtain the final models. In addition, twinning was accounted in the refinement of ligand-free YeGT structure. The quality of the structure was analyzed with MolProbity (53). For data collection and refinement statistics see Table 1. Structure figures were generated with Pymol, the Molecular Graphics System, Version 1.5.0.4 Schrödinger, LLC (www.pymol.com).

## Data availability

The atomic coordinates and structure factors of the ligand bound (code 8OVS) and of the ligand free (8OVT) YeGT were deposited in the Protein Data Bank (http://wwpdb.org/).

## Supporting information

This article contains supporting information.

## Conflict of interest

The authors declare that they have no conflicts of interest with the contents of this article.

## Supporting information

Supplemental Figures S1−S4 and Table S1
