# Supplementary material for: Tyrosine-modifying glycosylation by Yersinia effectors
Source: J Biol Chem. 2024 May 2;300(6):107331. doi: 10.1016/j.jbc.2024.107331 (PMC11152714; doi:10.1016/j.jbc.2024.107331)
Supplement: Supplemental Figures S1−S4 and Table S1 [file mmc1.docx]

**Supporting Information**

**Tyrosine modifying glycosylation by *Yersinia* effectors**

**Silvia Schneider^,1^, Christophe Wirth^2^, Thomas Jank^1^, Carola Hunte^2,3^, and Klaus Aktories^1,3^**

^1^ Institute for Experimental and Clinical Pharmacology and Toxicology, Faculty of Medicine, University of Freiburg, D-79104 Freiburg, Germany;

^2^ Institute for Biochemistry and Molecular Biology, ZBMZ, Faculty of Medicine, University of Freiburg, D-79104 Freiburg, Germany

^3^ Centre for Biological Signaling Studies (BIOSS), University of Freiburg, D-79106 Freiburg, Germany

**Table S1: Oligonucleotides**

|  | CACATGATCGCGATCGGTCCAAAGAATATTTC | QC YkGT W44A fw |
| --- | --- | --- |
|  | GAAATATTCTTTGGACCGATCGCGATCATGT | QC YkGT W44A rev |
|  | GTTTGCACAAGCAAGTGCCATTTTACGCC | QC YkGT D134A fw |
|  | GGCGTAAAATGGCACTTGCTTGTGCAAAC | QC YkGT D134A rev |
|  | CAAGTGACATTTTAGCCCTGTTGGTTC | QC YkGT R137 fw |
|  | GAACCAACAGGGCTAAAATGTCACTTG | QC YkGT R137 rev |
|  | GTATTTACAAGGCCATCGCTGCCATCCAAATTAAG | QC YkGT AxAA fw |
|  | CTTAATTTGGATGGCAGCGATGGCCTTGTAAATAC | QC YkGT AxAA rev |
|  | GGAGTTATGCGCGAGGCCGCGCCAAAGGCC | QC YkGT Y174A fw |
|  | GGCCTTTGGCGCGGCCTCGCGCATAACTCC | QC YkGT Y174A rev |
|  | GTTCCCAGAGGTGGCTGTGCCCACAG | QC RhoA Y34A fw |
|  | CTGTGGGCACAGCCACCTCTGGGAAC | QC RhoA Y34A rev |
|  | GAGGTGTATGCGCCCACAGTGTTTG | QC RhoA V35A fw |
|  | CAAACACTGTGGGCGCATACACCTC | QC RhoA V35A rev |
|  | GTGTATGTGGCCACAGTGTTTGAG | QC RhoA P36A fw |
|  | CTCAAACACTGTGGCCACATACAC | QC RhoA P36A rev |
|  | GTATGTGCCCGCAGTGTTTGAGAAC | QC RhoA T37A fw |
|  | GTTCTCAAACACTGCGGGCACATAC | QC RhoA T37A rev |
|  | GTGCCCACAGCGTTTGAGAACTATG | QC RhoA V38A fw |
|  | CATAGTTCTCAAACGCTGTGGGCAC | QC RhoA V38A rev |
|  | GTGCCCACAGTGGCTGAGAACTATG | QC RhoA F39A fw |
|  | CATAGTTCTCAGCCACTGTGGGCAC | QC RhoA F39A rev |
|  | CACAGTGTTTGCGAACTATGTGG | QC RhoA E40A fw |
|  | CCACATAGTTCGCAAACACTGTG | QC RhoA E40A rev |
|  | CAGTGTTTGAGGCCTATGTGGCAG | QC RhoA N41A fw |
|  | CTGCCACATAGGCCTCAAACACTG | QC RhoA N41A rev |
|  | GTTTGAGAACGCTGTGGCAGATATC | QC RhoA Y42A fw |
|  | GATATCTGCCACAGCGTTCTCAAAC | QC RhoA Y42A rev |
|  | GGCATATATAAAAACATTAATGATATAC | QC fw YeGT NxN |
|  | GTATATCATTAATGTTTTTATATATGCC | QC rev YeGT NxN |
|  | CCAATTTCCAGAAGTCGCCGTACCAACTGTCTTTGAAAAC | QC Rho1 Y39A fw |
|  | GTTTTCAAAGACAGTTGGTACGGCGACTTCTGGAAATTGG | QC Rho1 Y39A rev |
|  | GCCAATTTCCAGAAGTCTTCGTACCAACTGTCTTTGAAAACTATGTAGC | QC Rho1 Y39F fw |
|  | GCTACATAGTTTTCAAAGACAGTTGGTACGAAGACTTCTGGAAATTGGC | QC Rho1 Y39F rev |
|  | CCACATGATCGCGATCGGCCC | QC YeGT W44A fw |
|  | GGGCCGATCGCGATCATGTGG | QC YeGT W44A rev |
|  | CAAGCAAGTGCTATTCTGCGC | QC YeGT D134A fw |
|  | GCGCAGAATAGCACTTGCTTG | QC YeGT D134A rev |
|  | GTGATATTCTGGCCCTGCTGGTC | QC YeGT R137A fw |
|  | GACCAGCAGGGCCAGAATATCAC | QC YeGT R137A rev |
|  | CATGCGCGAGGCTGTGCCTGAAG | QC YeGT Y174A fw |
|  | CTTCAGGCACAGCCTCGCGCATG | QC YeGT Y174A rev |
|  | GCGAGTATGTGGCTGAAGCGGG | QC YeGT P176A fw |
|  | CCCGCTTCAGCCACATACTCGC | QC YeGT P176A rev |
|  | CCCAATAGCGCCATCGCAGCTAC | QC YeGT P188A fw |
|  | GTAGCTGCGATGGCGCTATTGGG | QC YeGT P188A rev |
|  | CTTGCCGGTGCAGATGTGTTCAC | QC YeGT P222A fw |
|  | GTGAACACATCTGCACCGGCAAG | QC YeGT P222A rev |
|  | GCTGATCACGCCTGGGCTACAG | QC YeGT S300A fw |
|  | CTGTAGCCCAGGCGTGATCAGC | QC YeGT S3000A rev |
|  | GCTGATCACAGCGCGGCTACAG | QC YeGT W301A fw |
|  | CTGTAGCCGCGCTGTGATCAGC | QC YeGT W301A rev |
|  | CAAAAATGGCCTGAGTCGACTCG | QC YeGT Y313A fw |
|  | CGAGTCGACTCAGGCCATTTTTG | QC YeGT Y313A rev |
|  | TTCAAGGATCCATGCAATATTTT | pET28a - YeGT fw (BamHI) |
|  | CTTGAGTCGACTCATGTAGCCCAGCTGTG | pET28a - YeGT C-10 rev (SalI) |
|  | CTTGAGTCGACTCATACATACTCGGAAAGACC | pET28a - YeGT C-20 rev (SalI) |
|  | CAAGCAAGTGCTATTCTGGCCCTGCTGGTC | QC YeGT D134A & R137A fw |
|  | GACCAGCAGGGCCAGAATAGCACTTGCTTG | QC YeGT D134A & R137A rev |
|  | GGAATCTACAAAGCTATCGCCGATATCCAG | YeGT+16 NxN fw |
|  | CTGGATATCGGCGATAGCTTTGTAGATTCC | YeGT+16 NxN rev |
|  | GCGCGAGTATGTGCCTAAAGCGGGGAAGTCAGC | YeGT E177K fw |
|  | GCTGACTTCCCCGCTTTAGGCACATACTCGCGC | YeGT E1771K rev |
|  | GCTGACGCTTCAAGAAATAGCGAAGATTTCACGCC | YeGT K276I fw |
|  | GGCGTGAAATCTTCGCTATTTCTTGAAGCGTCAGC | YeGT K276I rev |
|  | GAGTATGTATTGAATGGTGCTGATCACAGCTGG | YeGT C294L fw |
|  | CCAGCTGTGATCAGCACCATTCAATACATACTC | YeGT C294L rev |
|  | CTTGAGGATCCGAATTCATGCAGTACTTTACTCAG | YkGT-16 fw BamHI |
|  | TCAAGCTCGAGTCGACCTAGTACATTTTTG | YkGT-16 rev SalI |
|  | CAGCGAAGCGATGATTTTTG | pK92 Sequencing primer |

**Supplement Figure 1. Sequence alignment of glycosyltransferase toxins with secondary structure information of PaToxG (PDB code 4MIX).**

Sequence alignment: *Yersinia ruckeri* AfpP18 (Acc. No. C4ULG3), *Yersinia enterocolitica* YeGT (Acc. No. WP_139336879.1), *Yersinia kristensenii* YkGT (Acc. No. WP_049562392), *Photorhabdus asymbiotica* PaToxG (Acc. No. C7BKP9), *Clostridioides difficile* TcdB (Acc. No. P18177). The secondary structure elements are marked as coil for α-helices, as arrow for β-strands and as T for β-turns. Numbers at the beginning of the line indicate position in the different glycosyltransferase toxins. Strict identity is marked with a red box and the blue frame with red characters indicate group similarity. The alignment was created with ClustalW and the presentation was performed with ESPript (<http://espript.ibcp.fr>).


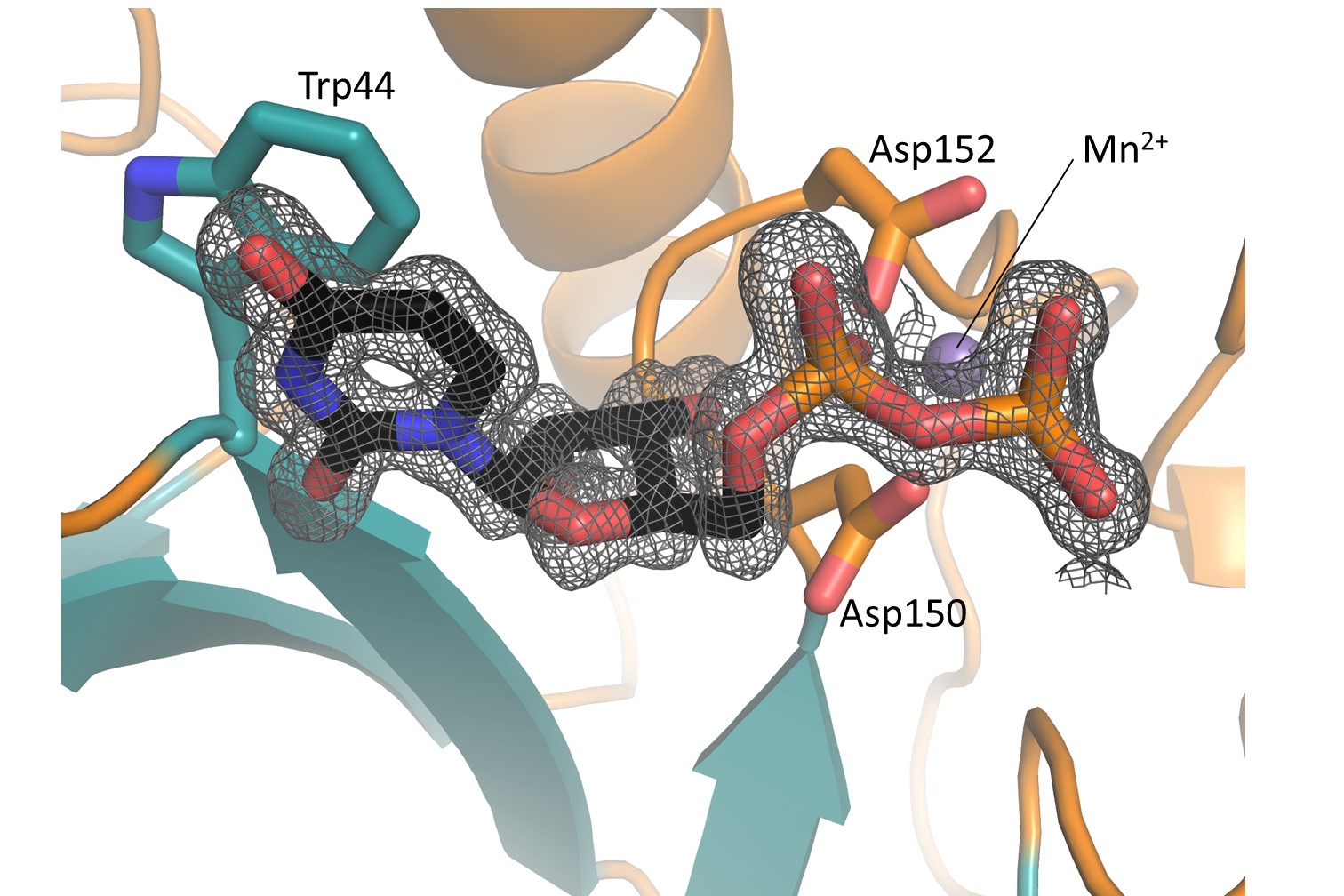


**Supplement Figure 2. Electron density map of the nucleotide in the UDP-bound sYeGT structure.**

View of the UDP (black) binding site in sYeGT, represented as cartoon and colored as in Figure 1, with key interacting residues represented as sticks. The 2Fo-Fc electron density map around the nucleotide was contoured at 1.6σ and represented as a grey mesh. Residues 125 to 135 were removed for clarity.


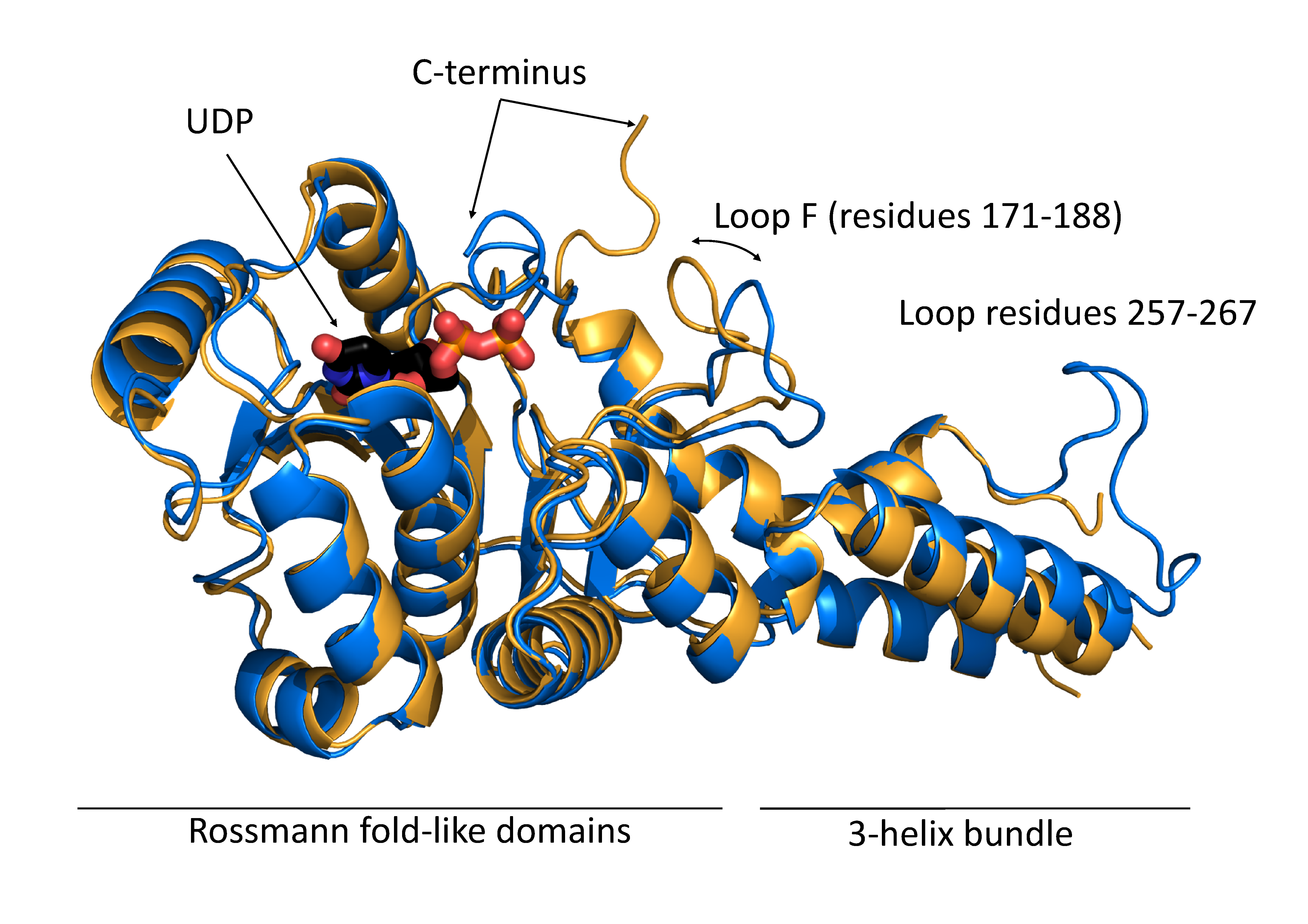


**Supplement Figure 3. Superimposition of ligand free and UDP-bound sYeGT structures.**

The structures of the ligand free sYeGT (blue) was superimposed onto the UDP-bound sYeGT structure (light orange) using SSM superimposition in Coot (51) and shown as cartoon representation. The Rossmann-like fold and the 3-helix bundle domains are specified. UDP is shown as sticks to highlight the substrate binding site. The largest variations observed between the two structures are indicated.


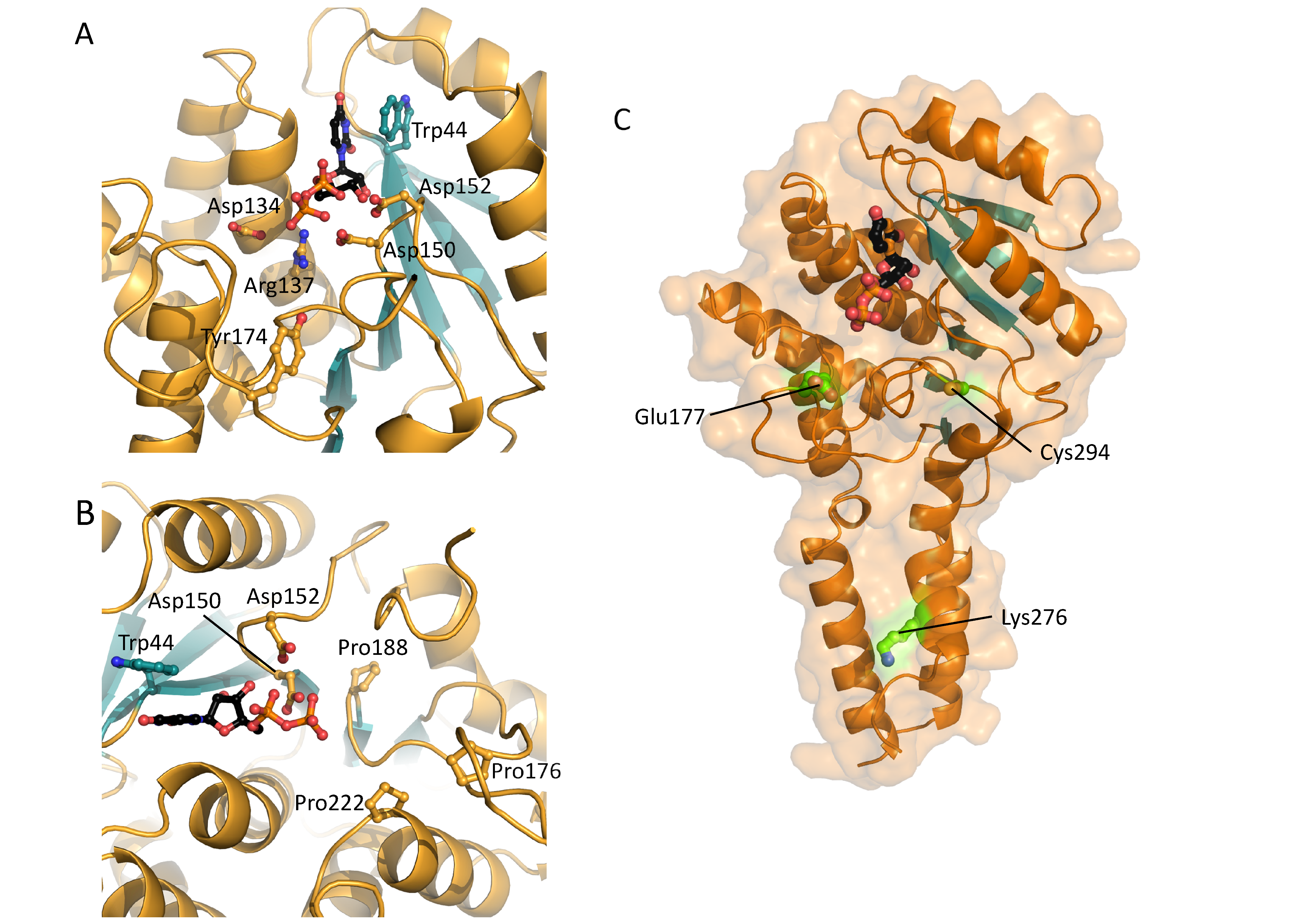


**Supplement Figure 4. Residues which were analyzed in the functional study of sYeGT.**

The UDP-bound sYeGT structure is represented in orange and teal, with the UDP molecule as well as residues of interest in sticks. (A) Residues analyzed to investigate their function in glycoside hydrolase and glycosyltransferase activities are indicated as well as the residues typical for glycosyltransferases (Trp-44, Asp-150, Asp-152). (B) The three proline residues present in or close to the flanking Loop F and that were investigated to analyze their impact on sYeGT function are displayed as well as key UDP coordinating residues. (C) Three residues that differ between sYeGT and YkGT and are located at the surface and on the side of the nucleotide binding site were analyzed and are represented in light green sticks. In order to show their surface exposition, a transparent surface of sYeGT is displayed.
